# Ammonium Sensing Patch with Ultrawide Linear Range and Eliminated Interference for Universal Body Fluids Analysis

**DOI:** 10.1007/s40820-024-01602-2

**Published:** 2024-12-23

**Authors:** Mingli Huang, Xiaohao Ma, Zongze Wu, Jirong Li, Yuqing Shi, Teng Yang, Jiarun Xu, Shuhan Wang, Kongpeng Lv, Yuanjing Lin

**Affiliations:** 1 School of Microelectronics, Southern University of Science and Technology, Shenzhen, 518055 People’s Republic of China; 2 Department of Applied Biology and Chemical Technology, The Hong Kong Polytechnic University, Hong Kong SAR, People’s Republic of China; 3 Department of Interventional Radiology, Shenzhen People’s Hospital, Shenzhen, 518020 People’s Republic of China; 4 College of Mechanical and Energy Engineering, Shaoyang University, Shaoyang, 422000 Hunan People’s Republic of China; 5 Shenzhen Hainwise Medical Technology Co., LTD, Shenzhen, 518118 People’s Republic of China; 6 Shenzhen Institute for Drug Control (Shenzhen Testing Center of Medical Devices), Shenzhen, 518000 People’s Republic of China; 7 Institute of Innovative Materials, Guangming Advanced Research Institute, Southern University of Science and Technology, Shenzhen, 518055 People’s Republic of China

**Keywords:** Biocompatible sensors, Implantable bioelectronics, Ammonium sensing, Cross-calibration, Ultrawide linear range

## Abstract

Ammonium level in body fluids serves as one of the critical biomarkers for healthcare, especially those relative to liver diseases. The continuous and real-time monitoring in both invasive and non-invasive manners is highly desired, while the ammonium concentrations vary largely in different body fluids. Besides, the sensing reliability based on ion-selective biosensors can be significantly interfered by potassium ions. To tackle these challenges, a flexible and biocompatible sensing patch for wireless ammonium level sensing was reported with an ultrawide linear range for universal body fluids including blood, tears, saliva, sweat and urine. The as-prepared biocompatible sensors deliver a reliable sensitivity of 58.7 mV decade^−1^ in the range of 1–100 mM and a desirable selectivity coefficient of 0.11 in the interference of potassium ions, attributed to the cross-calibration within the sensors array. The sensor’s biocompatibility was validated by the cell growth on the sensor surface (> 80%), hemolysis rates (< 5%), negligible cellular inflammatory responses and weight changes of the mice with implanted sensors. Such biocompatible sensors with ultrawide linear range and desirable selectivity open up new possibility of highly compatible biomarker analysis via different body fluids in versatile approaches.

**Supplementary Information:**

The online version contains supplementary material available at 10.1007/s40820-024-01602-2.

## Highlights

- The as-prepared sensors can detect NH_4_^+^ in the body fluids with a high sensitivity of 58.7 mV decade^−1^ and an ultrawide detection range of 1–100 mM.

- The biocompatible sensors exhibit desirable biocompatibility and minimal toxicity for continuous and long-term monitoring.

- The average detection error of the integrated and wireless biosensing patch was 13.2%, and body fluid detection accuracy is improved by more than 18% after cross-calibration.

**Supplementary Information:**

The online version contains supplementary material available at 10.1007/s40820-024-01602-2.

## Graphical abstract

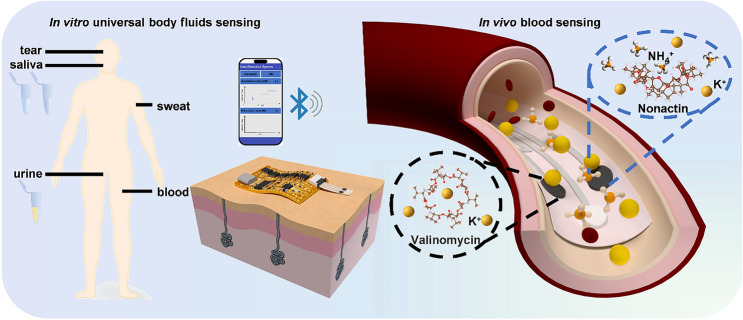

## Introduction

Ammonia is considered a byproduct of biological metabolism and is highly toxic to the central nervous system [1–6]. The pathophysiology of numerous illnesses, including hyperammonemia, chronic liver disease, hepatic encephalopathy, hepatocellular damage and Alzheimer's disease, is linked to variation in ammonium levels in body fluids [7–12]. Thus, it is imperative that ammonia in body fluids should maintain a low and stable concentration [13], and an efficient technique for convenient and continuous ammonia monitoring is highly demanded. Currently, colorimetric, photometric, enzymatic, gas-phase sensing, electrochemical impedance and potentiometric techniques are commonly adopted in detecting ammonia levels [14–25]. These methods typically involve invasive blood sensing via complicated procedures on the collected samples and cannot be performed in a continuous manner [26].

Alternatively, potentiometric ion-selective electrodes (ISEs) that can be integrated into wearable and portable platforms emerge as a cost-effective strategy for continuous and real-time monitoring. Since ammonia appears mostly in the form of NH_4_^+^ (around 99%) in the humoral environment of NH_4_^+^ in body fluids, ISEs based on nonactin ionophore for NH_4_^+^ tracking provide a promising solution [27, 28]. Nonactin is the most widely used ammonium ionophore in the past 20 years. Despite the significant interference from potassium, all-solid-state nonactin-based ISEs have been successfully applied in the fields of water quality assessment, clinical tests on biological fluids and sweat monitoring during sports practice (Table S1). Nevertheless, the NH_4_^+^ in body fluids shows a large variation in different body fluids ranging from several μM to tens of mM [29] (Table S2). Besides, the cross-interference arising from the similarity in ionic size and monovalent between K^+^ and NH_4_^+^ makes it challenging to accurately detect the actual NH_4_^+^ concentration in μM level, particularly in blood testing with relatively high K^+^ concentrations in mM level [30–32]. Thus, research efforts are expected to realize NH_4_^+^ ISEs with high sensitivity in ultrawide ranges, and cross-calibration of K^+^ concentration within the sensors array shows promise for the accurate determination of NH_4_^+^ concentrations.

Moreover, the biocompatibility of the sensors is crucial in the deployment of bioelectronics for wearable and implantable biomarker analysis in body fluids. For wearable applications, the sensing material should prioritize comfort and flexibility to ensure that the skin surface remains unharmed, while for implantable scenarios, the physical disparities between the sensing material and the inner wall of the blood vessel can result in inflammation, tissue damage, proliferation and other side effects [33–37]. Prior to approving the long-term wear and implantation of bioelectronics, it is imperative to ensure their compatibility with normal organism function and the absence of adverse reactions [38–42].

To achieve reliable NH_4_^+^ monitoring that is compatible with a variety of body fluids in both invasive and non-invasive manners, we developed a flexible and biocompatible patch consisting of NH_4_^+^ and K^+^ sensors array for wireless and universal ammonium sensing in body fluids, including tears, saliva, sweat, urine and blood. The sensing patch can detect NH_4_^+^ in the body fluids with a high sensitivity of 58.7 mV decade^−1^ and an ultrawide detection range of 1 μM -100 mM. The all-solid-state potential NH_4_^+^ and K^+^ sensors array possesses the cross-calibration capability to eliminate K^+^ interference with a favorable selectivity coefficient (K_ij_) of 0.11, thereby enabling accurate electrochemical signal output for ammonium sensing [28]. Moreover, the biocompatibility of the biosensors for long-term implantable applications has been evaluated both in vitro and in vivo. The cell growth rate is greater than 80% on the sensor surface and the hemolysis rate of each functional layer is less than 5%. Negligible cellular inflammatory responses and abnormal changes in body weight are also recorded for mice with implanted sensors. As a proof of concept, the NH_4_^+^ concentration in universal body fluids was assessed with the integrated wireless sensing system. The average detection error of the sensing system was evaluated to be around 13.2% and body fluid detection accuracy of the sensor is improved by more than 18% after cross-calibration, which indicates its desirable reliability. The as-developed integrated and wireless biosensing patch provides a promising strategy for real-time tracking of ammonium with ultrawide sensing range and highly desirable compatibility for universal body fluids analysis, which would no doubt shed light on the advances of bioelectronics for Internet of Medical Things (IoMT).

## Experimental Section

### Materials

Chromium (Cr), gold (Au), polytetrafluoroethylene (PTFE), UV glue (Weisite w-606), silver (Ag) paste, ferric (III) chloride (FeCl_3_), polyvinyl butyral resin BUTVAR B-98 (PVB), sodium chloride (NaCl), methanol, chloroauric acid (HAuCl_4_), hydrogen chloride (HCl), 3,4-ethylene dioxythiophene (EDOT), poly(sodium 4-styrenesulfonate) (NaPSS), potassium hexacyanoferrate(II) trihydrate (K_4_FeCN_6_), nonactin (ammonium ionophore), sodium tetrakis[3,5-bis(trifluoromethyl)phenyl] borate (Na-TFPB), high-molecular-weight polyvinyl chloride (PVC), Bis(2-ethylehexyl) sebacate (DOS), tetrahydrofuran (THF), valinomycin (potassium ionophore), sodium tetraphenylboron (NaTPB), cyclohexanone, PBS (Biosharp BL302A), ammonium chloride (NH_4_Cl), potassium chloride (KCl), calcium chloride (CaCl_2_), magnesium chloride (MgCl_2_), glucose, Calcein/PI Cell Viability/Cytotoxicity Assay Kit (C2015M, Beyotime Biotechnology, China), healthy C57BL/6 (male) mice of conventional grade (GEMPHARMATECH, Foshan, China), Masson's trichrome (C0189M, Beyotime Biotechnology, China), paraformaldehyde, paraffin, CCl_4_, ethanol, pentobarbital sodium, povidone iodine, Nylon, aqueous iodine. The artificial solution consists of amino acids: PBS mixed with 14 kinds of mice amino acids as shown in Table S3. All other chemicals were commercially available and used without further purification. All solutions were prepared using deionized water (16 M Ω cm) produced from a Millipore water purification system.

### Fabrication of Flexible NH_4_^+^ and K^+^ Sensors Array

A Cr/Au (thickness: 30 and 100 nm) pattern with three electrodes was designed and deposited on PTFE for the construction of NH_4_^+^ and K^+^ sensors via thermal evaporation (Thermal evaporation coating instrument DM300). The interconnects were then encapsulated by UV glue (ZhuoLiDe UV curving adhesive). Subsequently, a UV light with power of 100 W was employed for 15 s to cure the UV adhesive completely for encapsulation purposes. NH_4_^+^ and K^+^ sensors were constructed with a two-electrode configuration, where the Cr/Au electrodes were employed as building blocks for two working electrodes and one shared reference electrode. The shared Ag/AgCl reference electrode was fabricated by dispensing Ag onto the prepared Cr/Au pattern (Prtronic Scientific 3A), and followed by the etching process with 0.1 M FeCl_3_ for 60 s. 2 μL of PVB reference solution was then drop cast onto the Ag/AgCl layer to form Ag/AgCl/PVB reference electrode. The PVB reference was prepared by dissolving 79.1 mg PVB and 50 mg NaCl into 1 mL methanol. For the working electrode of the NH_4_^+^ and K^+^ sensors, the electrolyte for dendritic Au growth was a mixture of 50 mM HAuCl_4_ and 50 mM HCl. The deposition was conducted by applying a periodic voltage wave with an amplitude of -2 V, frequency of 50 Hz and duty cycle of 50% for 3000 cycles. This procedure was performed by the electrochemical workstations CHI660e with the electrochemical technique STEP (multi-potential steps). The applied parameters are as follows: step 1 voltage and time: 0 V and 0.01 s; step 2 voltage and time: -2 V and 0.01 s; No. of cycle: 3000. Poly(3,4-ethylenedioxythiophene) polystyrene sulfonate (PEDOT:PSS) was chosen as the ion–electron transducer and deposited onto the working electrodes. The electrolyte contains 10.6 μL EDOT, 206 mg NaPSS and 36.8 mg K_4_FeCN_6_ in 10 mL deionized (DI) water. PEDOT:PSS deposition was realized under a periodic voltage wave with an amplitude of 0.865 V (DC offset at 0.665 V with amplitude of 0.2 V), frequency of 1 Hz and duty cycle of 25% for 840 cycles. This procedure was performed by the electrochemical workstations CHI660e with the electrochemical technique STEP (multi-potential steps). The applied parameters are as follows: step 1 voltage and time: 0.465 V and 0.75 s; step 2 voltage and time: 0.865 V and 0.25 s; No. of cycle: 840. The NH_4_^+^ selective membrane cocktail consisted of nonactin (1% w/w), Na-TFPB (0.55% w/w), PVC (33% w/w) and DOS (65.45% w/w). 100 mg of the membrane cocktail was dissolved in 660 μL of THF. The K^+^ selective membrane cocktail was composed of valinomycin (2% w/w), NaTPB (0.5% w/w), PVC (32.7% w/w) and DOS (64.7% w/w). 100 mg of the membrane cocktail was dissolved in 350 μL of cyclohexanone. 6 μL of as-prepared NH_4_^+^ and K^+^ selective membrane cocktail was blade-coated onto the PEDOT:PSS-plated working electrodes. The sensor arrays were then dried overnight at 4 °C before use.

### Assembly of Flexible and Integrated Sensing System

The integrated sensing system was purchased from Shenzhen JLC Electronics Co., Ltd. The system adopted an integrated chip ESP32C3 as a microcontroller for multi-channel signal transmission and wireless display. The microcontroller for signal processing was programmed by the software Vscode. The potentiometric signal processing system mainly employed the LT1462ACS8 chip as a low-power operational amplifier. For the potentiometric signal processing, the input signal of the sensor at the millivolt (mV) level can be amplified with a gain factor of 3–5 that can be processed by the microcontroller using the amplifier. Then the microcontroller processed and transmitted the data to the phone via the Bluetooth chip. A lithium-ion battery of 100 mAh can directly power the whole system at a nominal voltage of 3.7 V.

### Characterization of Flexible NH_4_^+^ and K^+^ Sensors

Morphologies of samples were observed by the scanning electron microscope (ZEISS Gemini 300). The electrical properties of the samples under different bending states were investigated by a Keithley 2400 Sourcemeter coupled with a computer-controlled stretching motor. Electrodeposition and sensor performance were performed by the electrochemical workstations (CHI660e and Gamry reference 600 plus). As for sensors, direct recording of open-circuit potential (OCP) from the two-electrode system was adopted. The NH_4_^+^ and K^+^ concentrations in the collected samples were validated by ICP-MS (Thermo Q Exactive) and blood ammonia assay kit (Njjcbio A086-1–1).

### Interpolation Method

The NH_4_^+^ concentrations of body fluid samples were validated with the interpolation method. For ammonium results validation, the potential value of the ammonium sensor was first obtained in the solution with 0.01 mM ammonium solution. After drying, a certain amount of body fluid sample (50 μL) was applied to the sensors and the potential responses were recorded. The concentration of ammonium ions in the body fluid was then increased for a series of values by adding additional ammonium solution, and the corresponding responses were recorded. The slope k is obtained from the straight-line fitting of the relationship between the recorded sensor output potentials and the concentrations. The potential value of the solution without ammonium is *b*. The ammonium concentration in the original body fluid can be calculated:

1 $$ y = k\cdot\log x + b $$

Where *y* is the potential, and *x* is the concentration.

### Biocompatibility Evaluation of Ion-Selective Biosensors

#### In Vitro Cytotoxicity

The AM/PI staining experiment was conducted to investigate the sensor biocompatibility. Specifically, after being treated with ultraviolet sterilization, different parts (PTFE substrate, Au interconnect and ion-selective sensing electrode groups) of the sensor were placed into the 24-well plate; each group contained three repeated wells. Then HUVEC cells (the CellBank of Shanghai Institutes for Biological Sciences of the Chinese Academy of Sciences in China) were seeded in the wells with the sensor at a density of 1 × 10^4^ cells/well (500 μL/well), while the incubating condition is 5% CO_2_ and 37 °C. After 48 h, the AM/PI staining steps were carried on as follows: By the instructions of Calcein/PI Cell Viability/Cytotoxicity Assay Kit, the cells were gently washed with PBS three times; 250 μL Calcein AM/PI working fluid (AM 1X and PI 1X) was added to each well. After incubation at 37 °C for 20 min, the cells were observed and photographed through laser scanning confocal microscope (Leica, TCS SP8) of cell imaging.

#### Hemocompatibility

The in vitro hemolysis of different sensor layers including the UV glue, PTFE, Cr/Au, dendritic Au, PEDOT:PSS and ion-selective membrane (ISM) was determined by hemoglobin released from erythrocyte when the above layers were in direct contact with blood. Healthy C57BL/6 (male) mice of conventional grade (GEMPHARMATECH, Foshan, China) aged 8 weeks were adopted to collect blood. The blood was collected by picking out the eyeball with an anticoagulant tube. Four times the volume of sodium chloride (NaCl) injection was added into the fresh anticoagulant blood to prepare erythrocyte suspension. The suspension was centrifuged at 1500 rpm for 15 min, and the supernatant was removed. The precipitated red blood cells were washed and centrifuged two times with NaCl injection to obtain red blood cells. In the experimental group (UV glue, PTFE, Cr/Au, dendritic Au, PEDOT:PSS and ISM), 5 disks and 5 mL NaCl injection (twice the mass of the sample) were added into the test tube. In the negative control group (NC group), a 10 mL NaCl injection was added to the test tube. In the positive control group, 10 mL of DI water was added to the test tube. All the test tubes were immersed in a thermostatic water bath at 37 °C for 30 min, and then 0.2 mL of red blood cells was added into the test tubes. After blending, the test tubes were immersed in a thermostatic water bath at 37 °C for 60 min continually. Finally, the solution in the test tubes was collected and centrifuged at 1500 rpm for 5 min, and supernatant fluid was removed into the cuvette. The OD of the supernatant fluid was quantified photometrically using a Victor1420 (PerkinElmer Company, USA) at the wavelength of 570 nm. The test was replicated three times. Hemolytic properties were determined by the hemolysis ratio. The hemolysis ratio (Z) was calculated using the following equation:

2 $$ Z\left( \% \right) = \left( {(OD_{t} - OD_{nc} ) \times \left( {OD_{pc} - OD_{nc} } \right)} \right) \times 100\% $$

OD_t_, OD_nc_ and OD_pc_ refer to the average values of the measured optical density of the experimental group, negative control group and positive control group, respectively.

#### In Vivo Cytotoxicity

After 4 weeks of implantation, the mice were euthanized by overdose anesthesia. The heart, liver, spleen, lung and kidney were harvested, and the implanted samples with surrounding soft tissue were cut together. For the control group, the surgical point was taken as the center, and the surrounding tissue was taken. All the samples were fixed in 4% paraformaldehyde for about one day and then embedded in paraffin. The processed specimens' sections were stained with Hematoxylin/Eosin (HE) and Masson's trichrome staining. The histological images of fibrous capsulation around implants were observed and captured with a fully automatic inverted fluorescence microscope (Dmi8 + DFC7000T, Leica, Germany).

### Liver Cirrhosis Modeling

Specific pathogen-free (SPF) grade mature and healthy male Sprague-Dawley (SD) mice weighing from 300 to 320 g were purchased from GEMPHARMATECH (Foshan, China) and maintained at Shenzhen People's Hospital Translational Medicine Collaborative Innovation Center (Shenzhen, China). Subcutaneous injection of a 50% CCl_4_ soybean oil solution at 3 mL kg^−1^ body weight with an initial dose of 5 mL kg^−1^ body weight every 4 days was administered. Starting from the 5th injection, it was switched to intramuscular injection. A total of 15 injections were administered over 60 days. During the modeling process, animals were provided with a 10% ethanol solution instead of water and were fed a standard pellet diet. The general activity of the animals was observed daily, and their body weight was measured weekly. After the modeling process, whole blood was drawn for serum preparation and analysis. The liver, spleen and other organs were dissected for histopathological analysis using HE staining.

### In Vitro Body Fluid Analysis

The in vitro body fluids sensing was conducted by collecting the subject's naturally flowing tears, saliva, urine and exercise-induced sweat collected from the arms near the epidermal areas. The tear samples were collected with glass microcapillary tubes (LUOBENDE, China) at inner canthus of the eyelid or external canthus. The time needed for tear collection was up to 5 min. Saliva collection was performed after rinsing the mouth with water for one minute to eliminate contamination and stimulate the salivary glands. The saliva samples were collected with chewing sterile cotton rolls (Salivette at Sardest, Germany) for one minute in the mouth. The samples were immediately centrifuged at 1800 rpm for 15 min after collection. Sweat sensing was conducted with exercise-induced sweat collected from the subjects’ foreheads with microtubes every 400 s. The areas were wiped and cleaned with gauze before sample collection. About 4 mL of urine was taken from healthy volunteers and stored in a tube with lid. Four types of body fluid samples were collected with microtubes and wiped clean with gauze after sample collection. The body fluid samples were temporarily stored in the refrigerator at -4 °C for further ICP-MS and standard blood ammonia assay kit validation.

### In Vivo Mice Blood Analysis

Specific pathogen-free (SPF) grade mature and healthy C57BL/6 (male) mice weighing about 20 g were purchased from GEMPHARMATECH (Foshan, China) and maintained at Shenzhen People's Hospital Translational Medicine Collaborative Innovation Center (Shenzhen, China). Implantation was conducted at the gluteal muscle of a C57BL/6 mouse using a well-established and previously reported surgical method. Before surgery, the C57BL/6 mice were housed for 1 week for acclimatization. After that, all the C57BL/6 mice were placed on an operating table, and 45 mg kg^−1^ (2% w/v) pentobarbital sodium (Merck, Darmstadt, Germany) was provided as an anesthetic via intraperitoneal injection. After successful anesthesia, the hair of the surgical area was shaved and sterilized with 10% povidone iodine. Then, the NH_4_^+^ sensor was implanted into the mouse's abdomen to detect the blood from lacerated mesenteric capillaries. During in vivo blood sensing, the mouse was injected with antithrombin (BD vacutainer Lithium Heparin) to prevent blood clotting. The NH_4_^+^ sensor disks (Ø2 mm) were implanted separately into each mouse's abdomen. The subcutaneous tissue and skin were sutured with 4–0 Nylon, and the wound was sterilized with aqueous iodine. After implanting surgery, postoperative mice were separated to be fed with a regular diet and the healing of the surgical incision was regularly observed.

## Results and Discussion

### Design and Fabrication of the Ammonium Sensing Patch

**Fig. 1:**
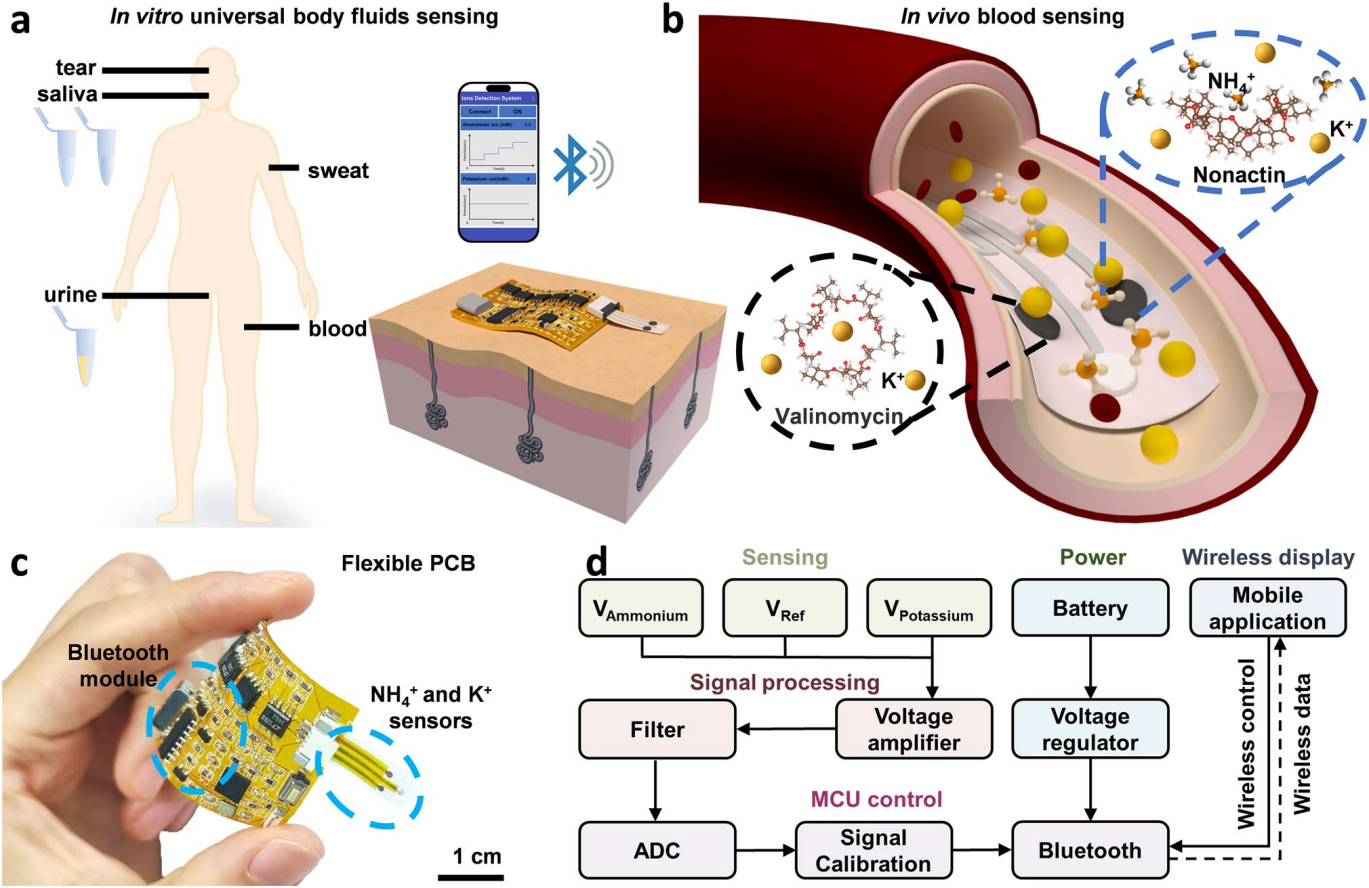
Schematic illustration and image of the integrated and wireless biosensing patch. **a** Schematic illustration of an ammonium sensing patch for wireless and in vitro body fluid analysis. **b** Schematic illustration of in vivo blood ammonia detection and competition of ion binding between NH_4_^+^ and K^+^ to nonactin. **c** Photograph showcasing the flexible PCB design. **d** Logical flow of the systematic design includes sensing, signal processing, MCU control, power supply and wireless display

The integrated and wireless biosensing patch was designed to be employed for NH_4_^+^ and K^+^ sensing in a large variety of body fluids. With systematic optimization on the active materials mass loading and layer architecture, the NH_4_^+^ sensors exhibit a broad detection range for in vitro analyzing various body fluids, including tears, saliva, sweat and urine (Fig. 1a). While nonactin was the most commonly utilized ionophores to construct selective NH_4_^+^ sensors, the interference from K^+^ with similar ionic radii remains a critical challenge for reliable sensing [32, 43]. Attributed to the self-calibration capability within the NH_4_^+^ and K^+^ sensors array, blood analysis with low ammonium levels can be achieved, which could otherwise be significantly interfered with the high potassium level (Fig. 1b). The flexible sensor arrays were integrated with a flexible printed circuit board (PCB) for real-time signal processing and wireless transmission via a Bluetooth module for mobile visualization (Figs. 1c and S1). The schematic diagram of the circuit design and the flowchart of the ammonium sensing patch are illustrated in Figs. 1d and S2. The extracted analog sensing signals were firstly transferred to the signal filter and voltage amplifier modules to match with the relatively high input impedance of the sensors. The output signals were converted into digital format through the analog-to-digital converter (ADC) and delivered to the microcontroller unit (MCU) for further calibration and transmission. The Bluetooth module then facilitated wireless data transmission to a user-friendly mobile display.

### NH_4_^+^ and K^+^ Interference Study and Decoding Method

**Fig. 2:**
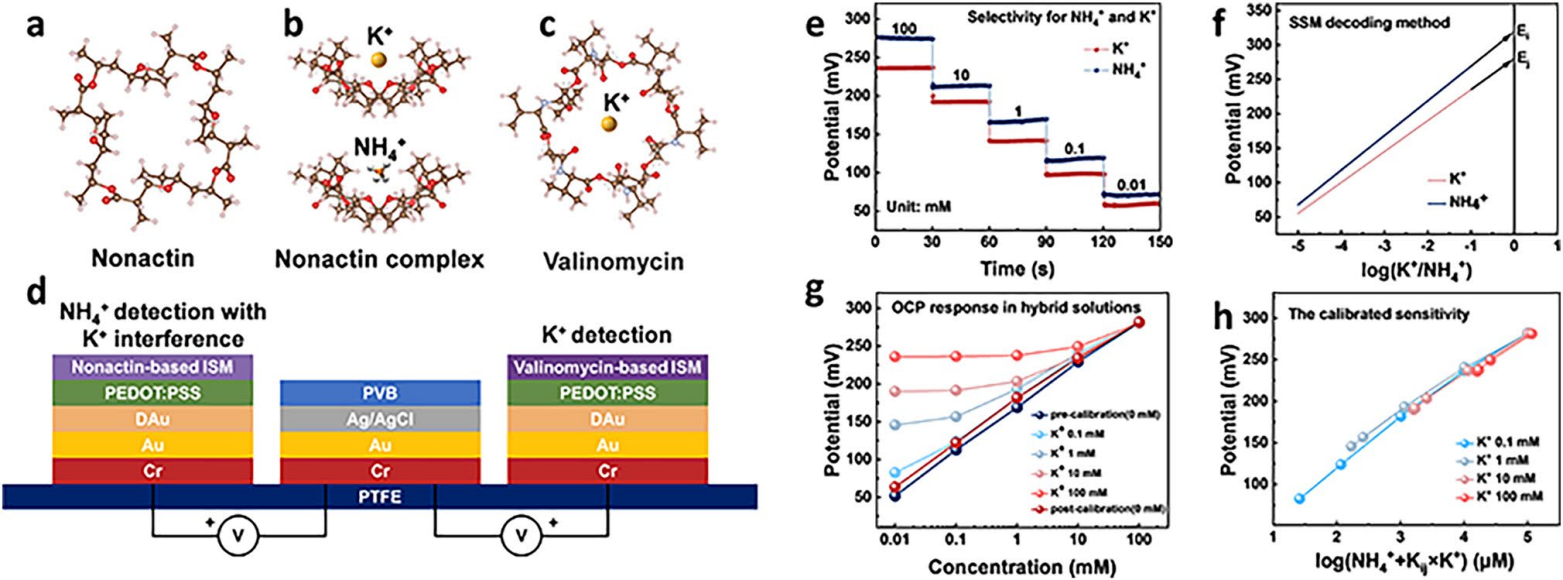
NH_4_^+^ and K^+^ interference study and decoding method. **a** Three-dimensional (3D) chemical structure of NH_4_^+^ ionophore (Nonactin). **b** Nonactin binding to K^+^ and NH_4_^+^, respectively. **c** 3D chemical structure of K^+^ ionophore (Valinomycin). **d** Structural design of the NH_4_^+^ and K^+^ ion-selective sensors based on two-electrode configuration with a shared reference electrode. **e** OCP responses of the NH_4_^+^ sensor to different concentrations of NH_4_^+^ and K^+^. **f** Illustration of the SSM decoding method. The selectivity coefficient is determined from the ${E}_{i}^{0}$ values, which can be obtained from extrapolation to $\text{log}{a}_{i}$=0. **g** OCP responses of NH_4_^+^ sensor in hybrid solutions with different concentrations of K^+^ as interfering ion. **h** Calibrated sensitivity with an average slope of 55.95 mV decade^−1^ with the SSM decoding method

The all-solid-state NH_4_^+^ sensors based on nonactin ionophore exhibit similar NH_4_^+^ and K^+^ selectivities (i.e., ${K}_{ij}^{pot}$ ranging from 0.04 to 0.16) [30]. It indicates when the value of K^+^ concentration is ten or twenty times higher than the NH_4_^+^ concentration, the NH_4_^+^ sensing accuracy can be largely interfered. Table S4 shows the similar ionic radii of NH_4_^+^ in 1.43 Å and K^+^ in 1.38 Å, compared to other majority interferences biomarkers in body fluids. Therefore, they possess comparable binding affinity to the spherical structure of nonactin (Fig. 2a, b). Compared with nonactin, valinomycin serves as the ideal ionophore for highly selective K^+^ sensing [44] (Fig. 2c). The NH_4_^+^ and K^+^ sensors in two-electrode configuration with a shared reference electrode were designed and fabricated on a PTFE substrate (Fig. 2d). The evaporated Au electrode patterns were texturized with dendritic Au that can provide large surface area (Fig. S3a-c). The ion-selective electrodes for both NH_4_^+^ and K^+^ were functionalized by conformably electroplating PEDOT:PSS as an ion-to-electron transducer (Fig. S3d) and dip-coating of the ionophore emulsions (Fig. S3e). The formation of the Cr/Au thin film, nanodendritic Au, PEDOT:PSS and ISM in each sensor layer is confirmed by energy-dispersive X-ray spectroscopy (EDX) and X-ray diffraction (XRD) analysis (Figs. S4 and S5). The shared reference electrode was functionalized with an Ag/AgCl/PVB layer to ensure voltage stability in solutions of varying ionic strengths. Afterward, UV glue was applied on the non-active sensing area for encapsulation to ensure the interconnect stability and conductivity in the liquid environment.

In order to decode the signals from the sensors array of NH_4_^+^ and K^+^, the separate solution method (SSM) was utilized to evaluate potentiometric selectivity coefficients (${K}_{ij}^{pot}$) over relevant interfering ions [30]:

3 $$ K_{ij}^{pot} = 10^{{\frac{{E_{j}^{0} - E_{i}^{0} }}{{\frac{2.303RT}{{z_{i} F}}}}}} $$

where $2.303RT/{z}_{i}F$ is a constant equal to 59.2 mV at 25 °C, ${z}_{i}$ is the charge of the measured ion, ${E}_{i}^{0}$ and ${E}_{j}^{0}$ are the individual potentials extrapolated to 1 M activity for the ions i(NH_4_^+^) and j(K^+^). Figure 2e shows the NH_4_^+^ sensor response to NH_4_^+^ and K^+^ in the range of 10 μM and 100 mM. The actual constant $2.303RT/{z}_{i}F$ as the slope of the curve is determined to be 51 mV, which is the sensitivity of the NH_4_^+^ sensor toward NH_4_^+^. The calibration curves were derived and selectivity coefficients (${K}_{ij}^{pot}$) were calculated as shown in Fig. 2f. The selectivity coefficient equals 0.15, indicating that K^+^ ions result in a 0.15-fold response to the open circuit potential (OCP) than NH_4_^+^ at the same concentrations. To validate the decoding accuracy of SSM, artificial solutions with interfering ions of K^+^ in the concentrations of 0.1, 1, 10, and 100 mM were applied onto the NH_4_^+^ electrode. The OCP results of the NH_4_^+^ sensor would be increased due to interference from K^+^. It is found that when the interfering K^+^ concentrations exceed 10 times than NH_4_^+^, the response of the NH_4_^+^ sensor would be significantly affected, leading to decreased reliability (Fig. S6). The non-negligible K^+^ interferences in high concentrations can also be confirmed by the electrochemical impedance spectroscopy (EIS) of the sensors in mixed solutions of ammonium chloride (NH_4_Cl) and potassium chloride (KCl) with various concentrations (Fig. S7). It can be seen that within the relatively low ion concentrations, the charge transfer resistance between NH_4_^+^ sensors and NH_4_^+^ is significantly smaller than K^+^, while with increased ion concentrations in the two kinds of solutions, the charge transfer resistances on the NH_4_^+^ sensors in both solutions become similar, which indicates the increased ion competition of K^+^ binding to the NH_4_^+^ sensor.

The extracted correlations between the output potential and NH_4_^+^ concentrations with different K^+^ levels were then calibrated using the Nikolskii–Eisenman equation, as an extension of the Nernst Equation:

4 $$ EMF = E_{i}^{0} + \left( {2.303RT/z_{i} F} \right)\log \left( {a_{i} + K_{ij}^{pot} a_{j}^{{z_{i} /z_{j} }} } \right) $$

where ${z}_{i}$ is the charge of the measured ion, ${z}_{j}$ is the charge of the interfered ion, ${a}_{i}$ and ${a}_{j}$ are the ionic activities of i(NH_4_^+^) and j(K^+^) in a mixed solution, and $EMF$ is the output potential in the mixed solution. Figure 2h shows the decoding results of the values of $\text{log}({a}_{i}+{K}_{ij}^{pot}{a}_{j}^{{z}_{i}/{z}_{j}})$ and $EMF$ with a slope of 55.95 mV decade^−1^, which is close to the theoretical Nernst equation slope of 59.2 mV decade^−1^. Thus, the reliability of the cross-calibration method can be validated.

### Performance Characterizations of the NH_4_^+^ and K^+^ Sensors Array

To achieve high sensitivity with an ultrawide sensing range for universal body fluids analysis, the electrochemical sensors array or NH_4_^+^ and K^+^ sensing were systematically optimized and characterized. To enable high sensitivity, the Au electrode was firstly decorated with nanodendritic Au. The response signals were largely enhanced compared with the unstable OCP response for those in planer Au film electrodes (Fig. S8a). PEDOT:PSS as the ion–electron transducer was introduced to improve long-term stability and suppress signal drift. Therefore, an optimal mass loading is critical to ensure enhanced signal stability without sacrificing the sensitivity. Compared with different PEDOT:PSS layers, the layers electroplating with 840 cycles deliver the optimized sensitivity with minimized voltage drift (Fig. S8b).

**Fig. 3:**
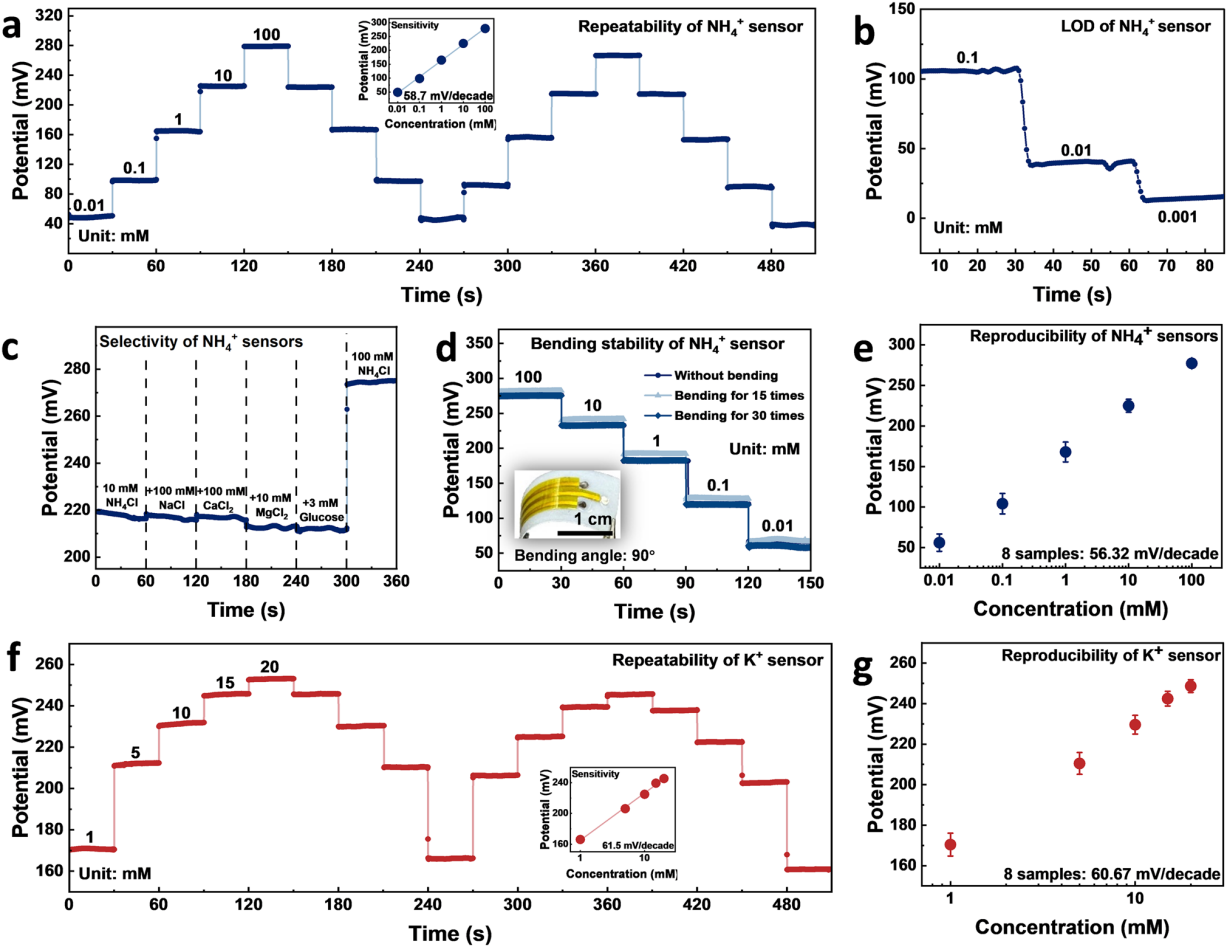
Characterization of the NH_4_ ^+^ and K^+^ ion-selective sensors. NH_4_^+^ sensor: **a** Ultra-large sensing range (10 μM -100 mM) with a sensitivity of 58.7 mV decade^−1^. **b** LOD for blood ammonium concentrations: 1 μM. **c** Selectivity apart from K^+^. **d** Bending stability. The inset of d shows the sensor after 30 cycles of bending. **e** Reproducibility of 8 NH_4_^+^ sensors. **f** Sensing performance of K^+^ sensor in the range of 1–20 mM with a sensitivity of 61.5 mV decade^−1^. **g** Reproducibility of 8 K^+^ sensors

The as-prepared NH_4_^+^ sensor delivered a sensitivity of 58.7 mV decade^−1^ and desirable repeatability from 10 to 100 mM, in which the concentration covers ammonium concentration in universal body fluids, such as sweat and blood (Fig. 3a). The sensors also exhibit a remarkable limit of detection (LOD) of 1 μM, and a desirable sensor drift down to 2.9 mV h^−1^ in 50 μM NH_4_Cl solution, which is the average blood NH_4_^+^ level (Figs. 3b and S9a-c). The selectivity of NH_4_^+^ sensor was investigated to ensure that the commonly seen interference biomarkers (such as NaCl, CaCl_2_, MgCl_2_, and glucose) in body fluids have limited effect on NH_4_^+^ monitoring (Fig. 3c). Additionally, the NH_4_^+^ sensor exhibits consistent sensing output even after multiple bending cycles and angles, demonstrating excellent flexibility and mechanical stability (Figs. 3d and S9d). Besides, Fig. 3e shows that the sensors exhibited good reproducibility with a relative standard deviation (RSD) of 11%. The performance, as observed from Table S1, is comparable to that of reported nonactin-based ISEs and superior to other ionophores apart from nonactin. Moreover, our universal body fluid tests, including blood samples with relatively low NH_4_^+^ levels, demonstrate competitive sensitivity and stability with a remarkable LOD and wide linear range for reliable applications in a variety of body fluids NH_4_^+^ sensing.

In addition, the as-prepared K^+^ sensor showed repeatability in the range of 1–20 mM for four cycles of testing with a sensitivity of 61.5 mV decade^−1^ (Fig. 3f). The linear response range covers/fulfills the sensing requirement 1.6–160 mM with reliable sensitivity (Fig. S10a). The selectivity was also investigated and it shows that the interference biomarkers (such as NH_4_Cl, NaCl, CaCl_2_, and glucose) have limited effect on K^+^ monitoring (Fig. S10b). Additionally, the K^+^ sensors exhibit similar output after bending cycles, demonstrating its desirable flexibility and mechanical stability (Fig. S10c). The sensor reproducibility was also assessed in different batches, yielding an average RSD of 10% (Fig. 3g). This confirms that the K^+^ sensor exhibits desirable sensitivity and stability for reliable sensing as well as calibration for both in vitro and in vivo monitoring scenarios.

### Biocompatibility Evaluation of the Ion-Selective Biosensors

**Fig. 4:**
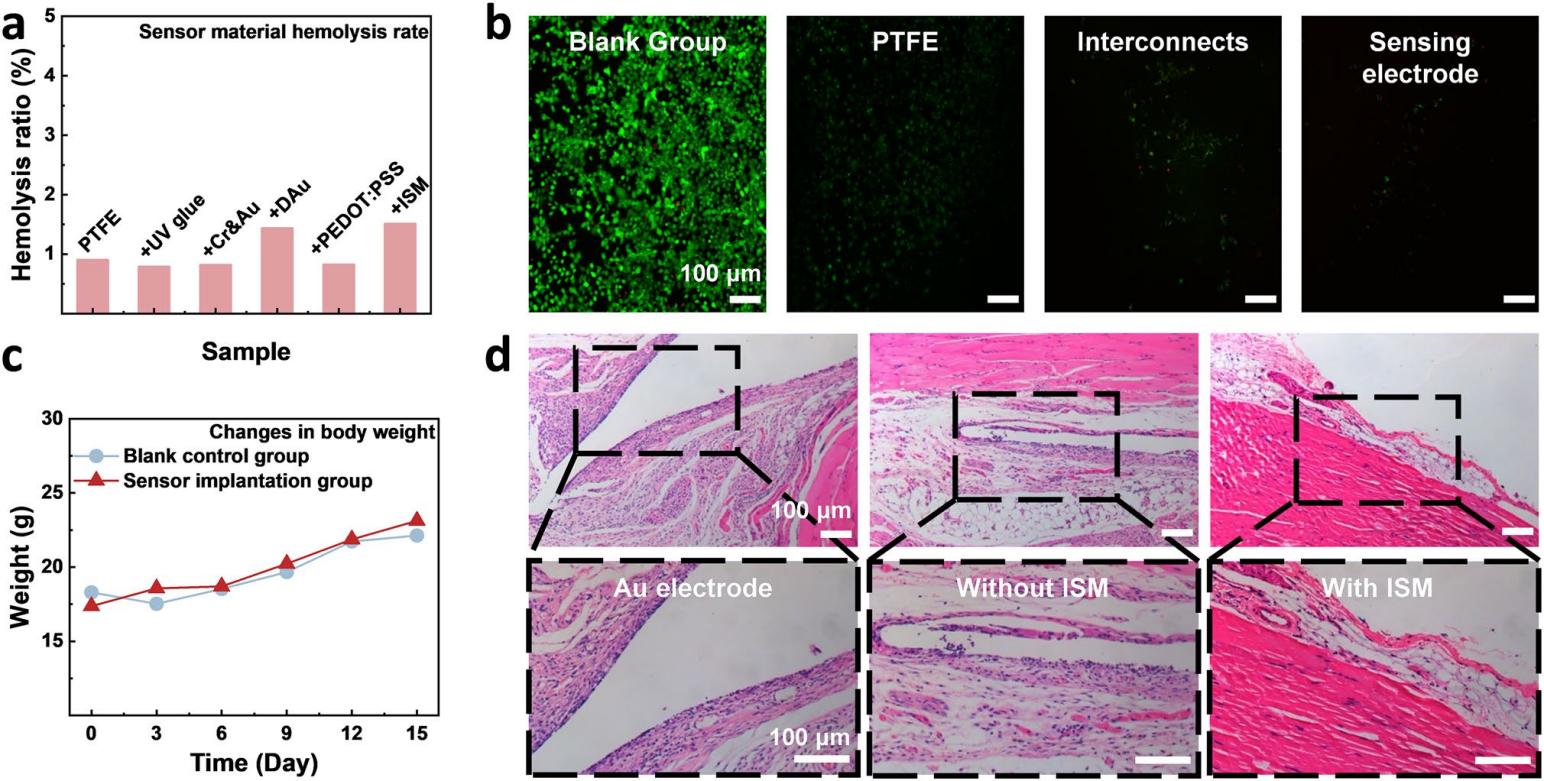
Biocompatibility evaluation of the ion-selective biosensors. **a** Hemocompatibility of different layers of the sensor. **b** In vitro cytotoxicity: live dead cells stained with AM/PI of the blank control group, experimental groups of PTFE substrate, Au interconnects and ion-selective sensing electrode. **c** Changes in the body weight of mice after subcutaneous implantation. **d** In vivo cytotoxicity: HE staining of implantation muscle tissue with implanted Au electrodes, which were encapsulated with UV glue, without ISM and with ISM group

Biocompatibility is necessarily required for long-term applications of bioelectronics. Hemocompatibility is the extent of rupturing of RBCs (erythrocytes) and burst release of hemoglobin from the inside out [45]. According to the experimental results, the hemolysis rate for each layer of the sensor is less than 2% (normally 5% complies with the hemocompatibility requirements), as shown in Fig. 4a. In terms of in vitro cytotoxicity, quantities of the live cells in the PTFE substrate, Au interconnect and ion-selective sensing electrode groups were similar to the blank control group, while the dead cells were rarely observed (Fig. 4b). The cell viability of the three experimental groups was greater than 80%. Moreover, the cell growth at the sensor electrode was significantly reduced, indicating the alleviated colonization of the cells and biomolecules on the sensor surface. Therefore, the risk of sensor failure due to cell accumulation can be avoided.

The subcutaneous implant experiment in mice was conducted to further examine the sensor's biocompatibility. The mice weights recorded at 0, 3, 6, 9, 12 and 15 days after sensor implantation are depicted in Fig. 4c. Compared to the control group mice without implanted sensors, those with implanted sensors showed a similar and steady weight gain tendency over time, which proved that the implanted sensor would not elicit any inflammatory response or disrupt normal daily physiological activities. To evaluate the in vivo cytotoxicity of the sensor, the histological finding of HE staining of the perimuscular fiber envelope after the sensor implantation into the muscle is shown in Fig. 4d. The cells in the group with ISM all had a distinct contour and shape, suggesting that the sensor implantation resulted in few inflammations. Nevertheless, a noticeable reduction in the thickness of the fiber envelope was observed in the ISM group compared with other groups, accompanied by the presence of new blood vessels. This suggests that sensor implantation in the ISM group elicited a mild inflammatory response and that the regenerative state of the implanted tissue was not affected. Additionally, HE staining results suggest that the implantation did not induce significant damage to vital organs such as the heart, liver, spleen, lungs and kidneys (Fig. S11). Therefore, it can be safely concluded that the as-fabricated flexible sensors exhibit desirable biocompatibility and minimal toxicity for continuous and long-term monitoring, especially for in vivo applications such as blood ammonium tracking.

### Demonstration of Universal Body Fluids Analysis In Vitro and In Vivo

**Fig. 5:**
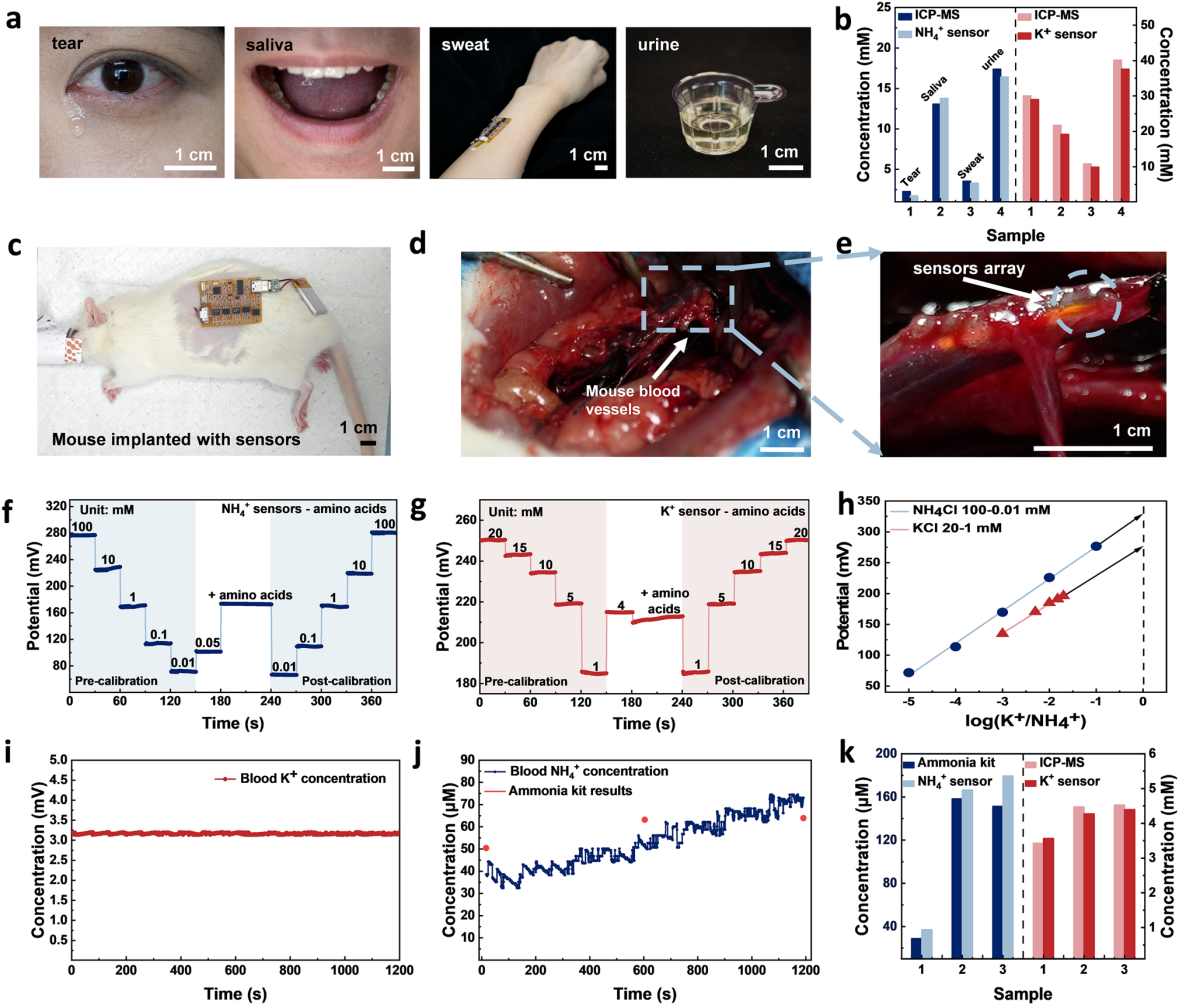
Ammonium analysis in universal body fluids with the biosensing patch. **a** Photographs of in vitro body fluid collection for tears, saliva, sweat and urine. **b** Representative NH_4_^+^ and K^+^ concentrations compared with standard validation. **c** Mice with sensing patch partially implanted into mouse abdomen. **d, e** Photograph of sensors array implanted in mouse blood vessels. **f** NH_4_^+^ sensor response to amino acid interference. **g** Amino acids impact for K^+^ sensor. **h** K_ij_ calculation via SSM decoding method. **i** Real-time in vivo K^+^ concentrations tracking for a healthy mouse. **j** Real-time in vivo NH_4_^+^ concentrations tracking for a healthy mouse with elimination of the amino acids and K^+^ interferences. **k** Representative serum NH_4_^+^ and K^+^ concentrations compared with standard validation. Serum 1 is from the control group in a healthy state, and Serums 2 and 3 are from experimental groups with liver cirrhosis

The as-developed integrated and wireless biosensing patch with ultrawide linear range and biocompatibility was demonstrated for in vitro and in vivo universal body fluids analysis. The system accuracy was also characterized by the OCP input of the sensor and output of signal processing module. The voltage amplification exhibited a high goodness of fit (*R*^2^ = 0.994), indicating the robustness and reliability of the biosensing patch for bio signal processing (Fig. S12a). The biosensing patch maintains stable sensing output after 100 different bending cycles and higher battery capacity provides higher patch working life since the patch uses Bluetooth low energy (BLE) as communication method, demonstrating the good flexibility and mechanical stability (Fig. S12b, c). Figures 5a and S13-S16 illustrate the in vitro detection of NH_4_^+^ and K^+^ in tears, saliva, sweat, and urine. The sensors were calibrated with an artificial solution before and after the on-body test to ensure their sensing reliability. The sensing accuracy was validated using commercial blood ammonia kits and inductively coupled plasma mass spectrometry (ICP-MS), yielding reliable results with a maximum RSD of 22.6% and 11.8% through cross-calibration (Fig. 5b).

While it is relatively straightforward to extract reliable sensing results for body fluids that can be collected non-invasively, it requires more careful considerations for in vivo blood sensing. To demonstrate in vivo blood sensing, the liver cirrhosis model was established in mice, and one serum sample from the control group and the other two from experimental groups with liver cirrhosis were collected (Fig. S17). The development of ulcerations, reduction in serum biochemical markers, and modulation of inflammatory responses in cells collectively confirmed the successful induction of cirrhosis in the model (Fig. S18). As a proof of concept, the sensing patch was partially mounted into the mice (Fig. 5c). Attributed to the flexibility and the miniaturized sensor sizes, the sensors array can be implanted within the femoral artery of the mice (Fig. 5d, e).

It should be noted that the intricate composition of blood would normally lead to imprecise measurements of blood ammonia concentration. For instance, the presence of unbound amino acids in plasma can form unstable associations with non-motorized proteins [46, 47]. Most of the organs have a relatively stable metabolism of proteins that recycle most amino acids over time and the concentrations of amino acids in serum normally remain at steady levels (Table S3). Additionally, it is reported that the concentrations of serum amino acids in healthy mice and mice with liver cirrhosis are similar (3.94 and 3.82 mM) [48]. Therefore, an artificial solution consisting of amino acids with a concentration of 3.82 mM was utilized to extract calibration factors of OCP responses to the amino acid. Figure 5f, g shows the interference effects of amino acids on NH_4_^+^ and K^+^ sensors, indicating the OCP response of the K^+^ sensor to amino acid was significantly smaller (3.12 mV), whereas the NH_4_^+^ sensor exhibited an increased response of 71.9 mV. To extract reliable in vivo blood ammonium levels, the interferences of amino acids and the competitive effect of K^+^ on the NH_4_^+^ sensor should be calibrated, based on the above interference factors from both amino acid and K^+^ with the selectivity coefficient *K*_ij_ = 0.11 (Fig. 5h).

For real-time in vivo demonstration, the sensors array was implanted into the mouse abdomen to detect the blood from lacerated mesenteric capillaries, whereas the NH_4_^+^ concentration can be continuously monitored and wirelessly displayed on the custom-designed mobile application (Fig. S19 and Movie S1). The real-time sensing results show that the K^+^ concentrations are around 3.2 mM and the NH_4_^+^ concentrations are 40–70 μM, which are in the reasonable ranges for a healthy mouse (Fig. 5i, j) and indicate the promising applications for in vivo biosensing. The decoded NH_4_^+^ concentrations extracted from the sensing patch display an average RSD of 21.4%. The fluctuation and an upward trend of ammonium ions could be attributed to several factors. Firstly, the inherent involvement of ammonia in body metabolism may lead to their inherent variability. Secondly, certain anesthetics can have an instant impact on liver function, nerves regulation and heart rate, which may slow down the metabolism rate of ammonia elimination rate of liver and therefore result in the rise of blood ammonium concentration. To further validate the sensing accuracy, the OCP responses of real-time blood NH_4_^+^ sensing can be calibrated from 3 different mice serum samples. The accurate K^+^ and NH_4_^+^ concentrations in the serums from mice in health and liver cirrhosis states can be calculated from the K^+^ sensor response (Fig. S20) and calibrated NH_4_^+^ response (Fig. S21) using the Nernst equation. The accuracy was evaluated using ICP-MS and commercial blood ammonia kits, indicating an average RSD of 3.78% for K^+^ and 17.39% for NH_4_^+^, respectively (Fig. 5k). The results obtained from standard analysis revealed that mice with liver disease exhibited comparable blood potassium concentrations to those of normal mice, while their blood ammonium levels were significantly elevated, reaching 144 μM which is around twice of the maximum concentration observed in healthy mice. In summary, the as-developed biosensing patch provides a promising strategy for real-time tracking of ammonium with a reliable sensitivity of 58.7 mV decade^−1^ in the ultrawide range of 1 μM -100 mM for universal body fluids analysis as summarized in Table S5. After eliminating the impact with amino acid and the cross-calibration with K^+^ interference, the relative errors of body fluids detection are below 22.6%, indicating the considerate reliability of such biocompatible ammonium sensing patches for the wide applications in wearable, portable and implantable monitoring. However, the long-term stability of the sensor was challenged by contamination and damage from the vein blood and interstitial fluid, including proteins, lipids, organic acids and other complex internal environment compositions, which hinders the sensor in vivo performance and its long-term application.

## Conclusions

Ammonium serves as one of the critical biomarkers for healthcare, while it poses a high requirement on the sensing ranges since the ammonium concentration in different body fluids can largely vary from μM to mM level. Besides, the reliability of ion-selective biosensors for ammonium sensing can be significantly compromised by the presence of K^+^ and free amino acids, posing a challenge in extracting reliable values for both in vitro and in vivo sensing scenarios. Herein, we present a flexible and biocompatible patch specifically designed for wireless and universal NH_4_^+^ monitoring to fulfill the clinical demand for reliable and convenient ammonium tracking. The as-prepared ammonium sensor showed an ultrawide linear range from 1 to 100 mM and a desirable sensitivity of 58.7 mV decade^−1^ for universal body fluids, including tears, saliva, sweat, urine, and blood. To achieve reliable ammonium detection in body fluids, especially for blood analysis with low NH_4_^+^ levels compared to other interference biomarkers, the cross-calibration of interfering K^+^ and elimination of amino acids impact were also investigated. In comparison to the validated results from analytical tools, the concentrations of NH_4_^+^ in a variety of body fluids determined via the proposed approach delivered an RSD of less than 22.6% and achieved an accuracy enhancement of more than 18% compared with non-calibrated results. Furthermore, the real-time, continuous and wireless NH_4_^+^ monitoring capability of the integrated sensing system was demonstrated via implantation into mice. The in vitro and in vivo experimental results demonstrated that the patch exhibited good biocompatibility, thereby expanding the potential applications of this implantable bioelectronics. The as-developed flexible and biocompatible sensing patch with ultrawide linear range demonstrated promising applications for universal body fluids analysis in both in vitro and in vivo scenarios. The proposed integrated biosensing system with cross-calibration within the sensors array would inspire the advances of bioelectronics that are capable of versatile applications in wearable, portable and implantable manners.

## Supplementary Information

Below is the link to the electronic supplementary material.

Supplementary file1 (DOCX 18 KB)

Supplementary file2 (DOCX 12917 KB)

Supplementary file3 (MP4 20444 KB)
