# Supplementary material for: Ammonium Sensing Patch with Ultrawide Linear Range and Eliminated Interference for Universal Body Fluids Analysis
Source: Nanomicro Lett. 2024 Dec 23;17:92. doi: 10.1007/s40820-024-01602-2 (PMC11663834; doi:10.1007/s40820-024-01602-2)
Supplement: Supplementary file 1 — Supplementary file1 (DOCX 18 KB) [file 40820_2024_1602_MOESM1_ESM.docx]

#include <Arduino.h>

#include <BLEDevice.h>

#include <BLEServer.h>

#include <BLEUtils.h>

#include <BLE2902.h>

#include <ESP32AnalogRead.h>

BLEServer *pServer = NULL;

BLECharacteristic * pTxCharacteristic;

bool deviceConnected = false;

bool oldDeviceConnected = false;

uint8_t txValue = 0;

const int ADC1_4 = 32;//

const int ADC1_5 = 33;//

const int ADC1_6 = 34;//

const int ADC1_7 = 35;//

ESP32AnalogRead adc_4;

ESP32AnalogRead adc_5;

ESP32AnalogRead adc_6;

ESP32AnalogRead adc_7;

// See the following for generating UUIDs:

// https://www.uuidgenerator.net/

#define SERVICE_UUID "C6FBDD3C-7123-4C9E-86AB-005F1A7EDA01" // UART service UUID

#define CHARACTERISTIC_UUID_RX "B88E098B-E464-4B54-B827-79EB2B150A9F"

#define CHARACTERISTIC_UUID_TX "D769FACF-A4DA-47BA-9253-65359EE480FB"

//

class MyServerCallbacks: public BLEServerCallbacks {

void onConnect(BLEServer* pServer) {

deviceConnected = true;

};

void onDisconnect(BLEServer* pServer) {

deviceConnected = false;

}

};

//

class MyCallbacks: public BLECharacteristicCallbacks {

void onWrite(BLECharacteristic *pCharacteristic) {

std::string rxValue = pCharacteristic->getValue();

if (rxValue.length() > 0) {

Serial.println("*********");

Serial.print("Received Value: ");

for (int i = 0; i < rxValue.length(); i++)

Serial.print(rxValue[i]);

Serial.println();

Serial.println("*********");

}

}

};

void setup() {

// put your setup code here, to run once:

adc_4.attach(ADC1_4);

adc_5.attach(ADC1_5);

adc_6.attach(ADC1_6);

adc_7.attach(ADC1_7);

Serial.begin(115200);

// Create the BLE Device

BLEDevice::init("ESP32_APP");

// Create the BLE Server

pServer = BLEDevice::createServer();

pServer->setCallbacks(new MyServerCallbacks());

// Create the BLE Service

BLEService *pService = pServer->createService(SERVICE_UUID);

// Create a BLE Characteristic

pTxCharacteristic = pService->createCharacteristic(

CHARACTERISTIC_UUID_TX,

BLECharacteristic::PROPERTY_NOTIFY

);

pTxCharacteristic->addDescriptor(new BLE2902());

BLECharacteristic * pRxCharacteristic = pService->createCharacteristic(

CHARACTERISTIC_UUID_RX,

BLECharacteristic::PROPERTY_WRITE

);

pRxCharacteristic->setCallbacks(new MyCallbacks());

// Start the service

pService->start();

// Start advertising

pServer->getAdvertising()->start();

Serial.println("Waiting a client connection to notify...");

}

void loop() {

// put your main code here, to run repeatedly:

if (deviceConnected) {

float x = adc_4.readVoltage()*100;//

float x1 = adc_5.readVoltage()*100;//

float x2 = adc_6.readVoltage()*100;//

float x3 = adc_7.readVoltage()*100;//

//analogRead(ADC)/4096.0*3.3;//

char txString[8];//

char txString1[8];//

char txString2[8];//

char txString3[8];//

dtostrf(x, 1, 2, txString);//

dtostrf(x1, 1, 2, txString1);//

dtostrf(x2, 1, 2, txString2);//

dtostrf(x3, 1, 2, txString3);//

char s[]="";

strcat(s,txString);

strcat(s," ");

strcat(s,txString1);

strcat(s," ");

strcat(s,txString2);

strcat(s," ");

strcat(s,txString3);

pTxCharacteristic->setValue(s);//

pTxCharacteristic->notify();//

Serial.print("*** Sent Value: ");

Serial.print(x3);

Serial.println(" ***");

}

delay(2000); // bluetooth stack will go into congestion, if too many packets are sent

// disconnecting

/*

if (!deviceConnected && oldDeviceConnected) {

delay(500); // give the bluetooth stack the chance to get things ready

pServer->startAdvertising(); // restart advertising

Serial.println("start advertising");

oldDeviceConnected = deviceConnected;

}

// connecting

if (deviceConnected && !oldDeviceConnected) {

// do stuff here on connecting

oldDeviceConnected = deviceConnected;

}

*/

}
