# Supplementary material for: Ammonium Sensing Patch with Ultrawide Linear Range and Eliminated Interference for Universal Body Fluids Analysis
Source: Nanomicro Lett. 2024 Dec 23;17:92. doi: 10.1007/s40820-024-01602-2 (PMC11663834; doi:10.1007/s40820-024-01602-2)
Supplement: Supplementary file 2 — Supplementary file2 (DOCX 12917 KB) [file 40820_2024_1602_MOESM2_ESM.docx]

**Ammonium Sensing Patch with Ultrawide Linear Range and Eliminated Interference for Universal Body Fluids Analysis**

Mingli Huang^1^, Xiaohao Ma^1^, Zongze Wu^2^, Jirong Li^2,3^, Yuqing Shi^1^, Teng Yang^2^, Jiarun Xu^4^, Shuhan Wang^5^, Kongpeng Lv^2, 6,^ * and Yuanjing Lin^1,^ *

^1^ School of Microelectronics, Southern University of Science and Technology, Shenzhen 518055, China

^2^ Department of Interventional Radiology, Shenzhen People’s Hospital, Shenzhen 518020, China

^3^ College of Mechanical and Energy Engineering, Shaoyang University, Shaoyang, Hunan 422000, China

^4^ Shenzhen Hainwise Medical Technology Co., LTD, Shenzhen 518118, China

^5^ Shenzhen Institute for Drug Control (Shenzhen Testing Center of Medical Devices), Shenzhen 518000, China

^6^ Institute of Innovative Materials, Guangming Advanced Research Institute, Southern University of Science and Technology, Shenzhen 518055, China

*Corresponding author. E-mail: kp.lv@foxmail.com; linyj2020@sustech.edu.cn


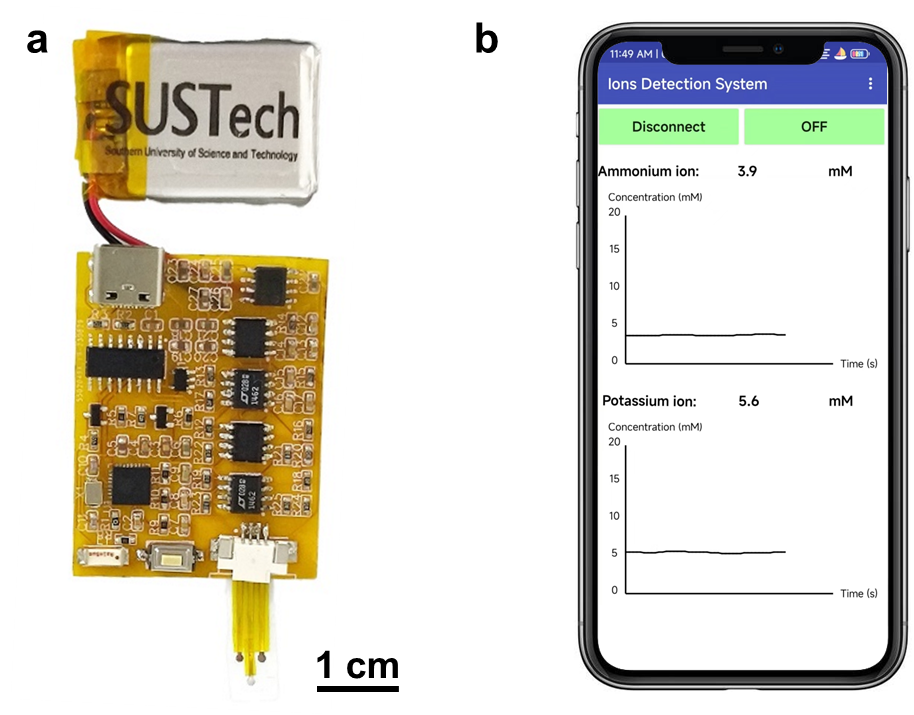


**Supplementary Fig. 1. The sensing system with battery and its wireless display via mobile phone App. a** The image of the sensing system includes sensors, flexible PCB, battery and **b** Wireless display via cellphone App.


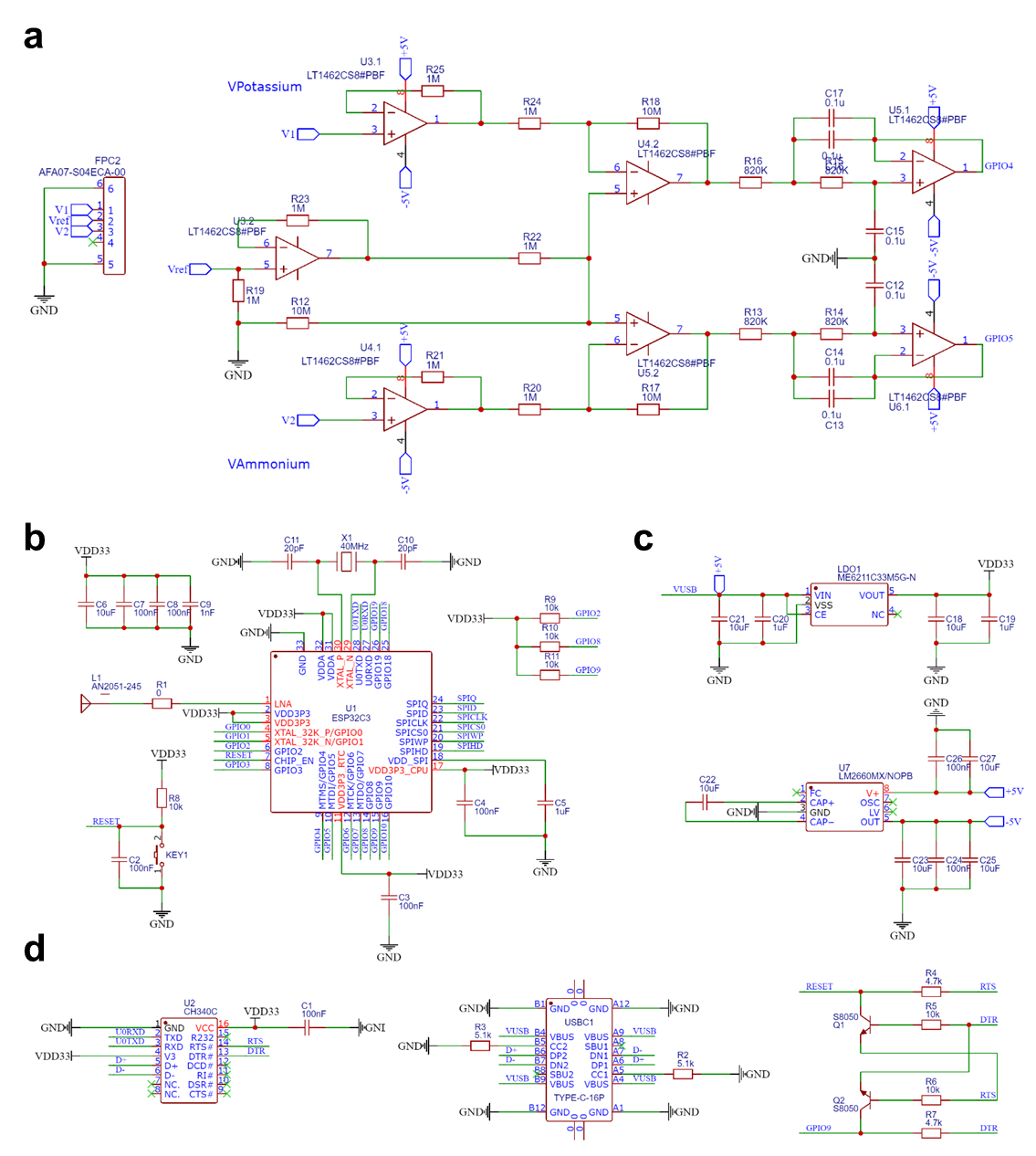


**Supplementary Fig. 2. Schematic design of the sensing system. a** Signal conditioning module. **b** MCU control module. **c** Power management module. **d** Programming module.


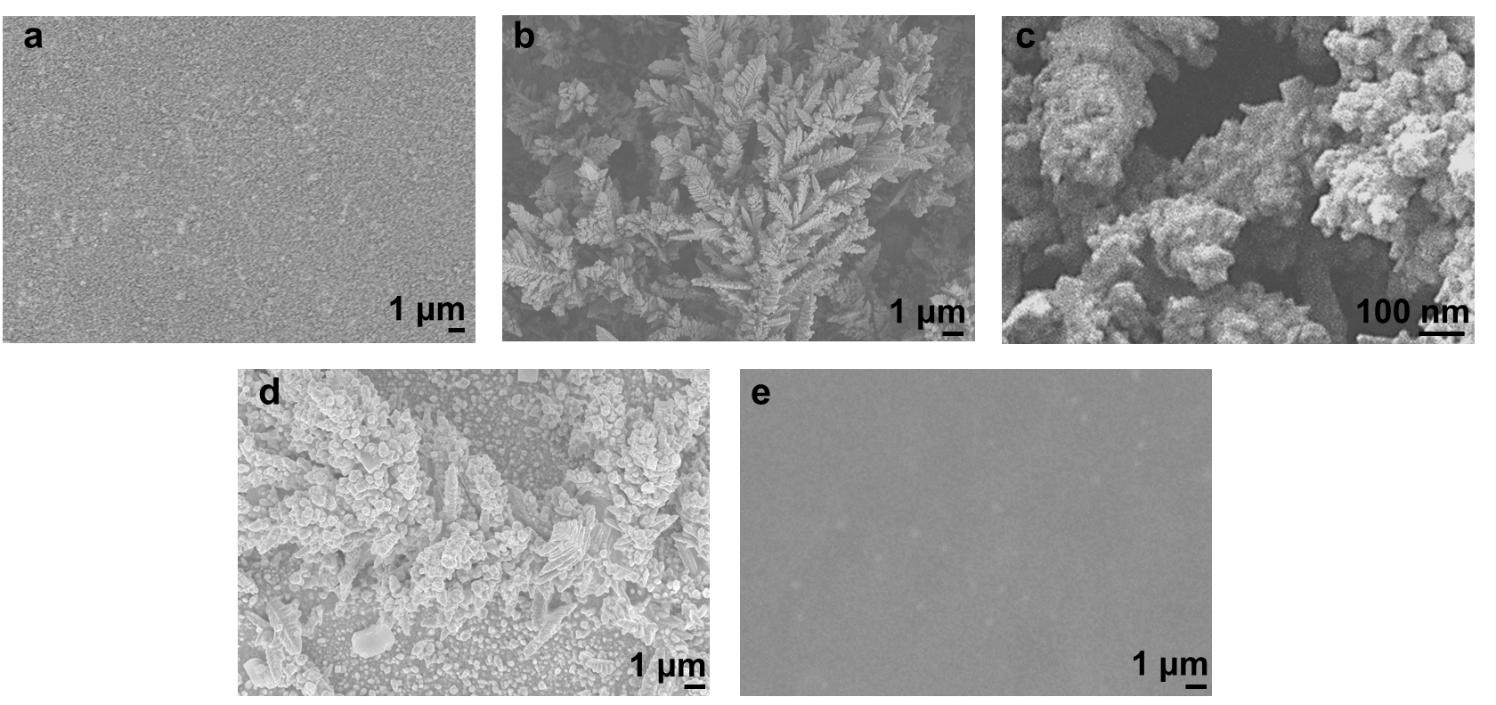


**Supplementary Fig. 3. SEM characterization of different layers for the NH_4_^+^ sensor.** **a** Au electrode. **b&c** Dendritic Au at X3000 and X20000 magnifications. **d** PEDOT: PSS on dendritic Au. **e** Selective membrane.


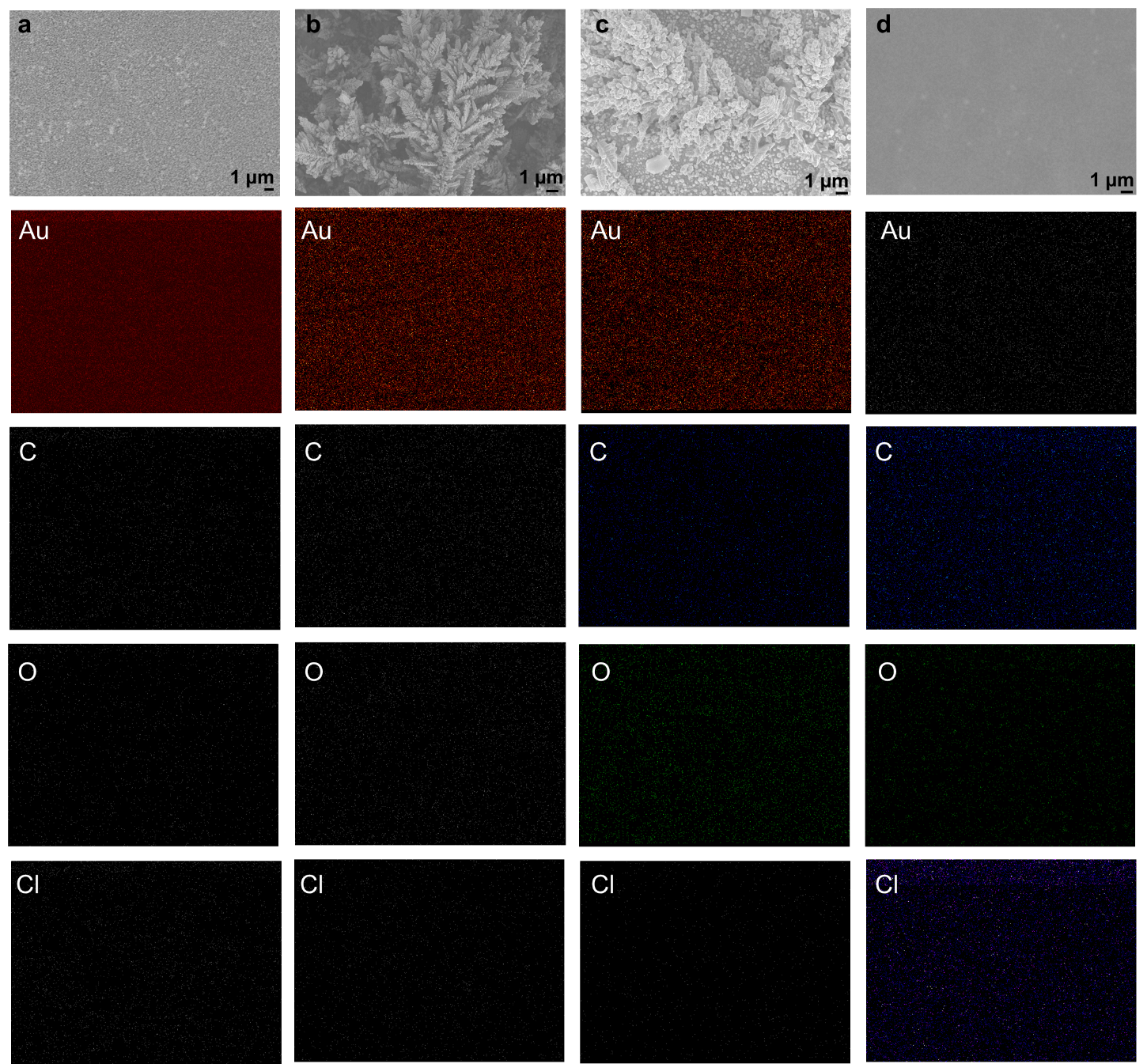


**Supplementary Fig. 4. EDX characterization of different layers for the NH_4_^+^ sensor. a** Au electrode with Au element. **b** Dendritic Au with Au element. **c** PEDOT: PSS on dendritic Au with C and O elements. **d** Selective membrane with C, O and Cl elements.

**Supplementary Fig. 5. XRD characterization of different layers for the NH_4_^+^ sensor.**


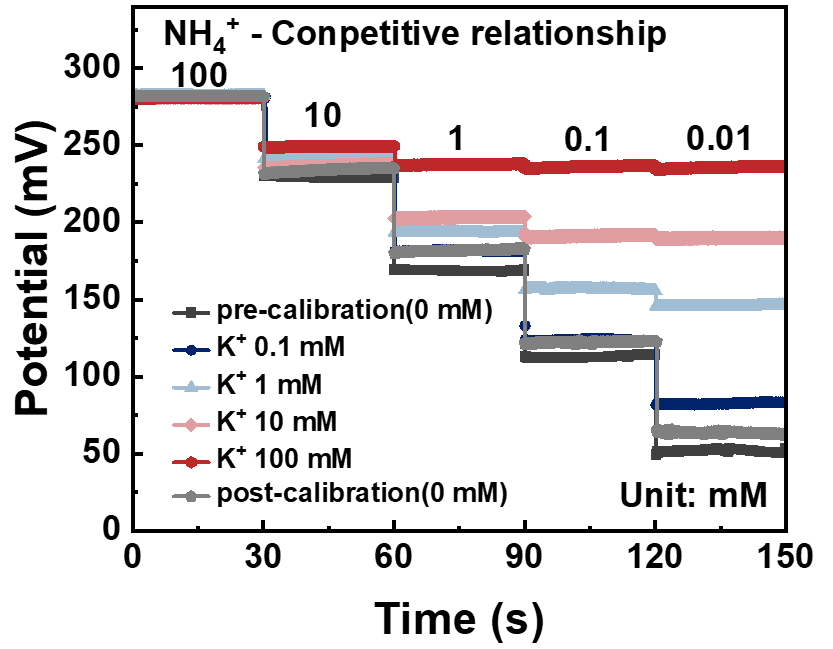


**Supplementary Fig. 6. NH_4_^+^ sensor hybrid response with different concentrations of K^+^ as an interfering ion.**


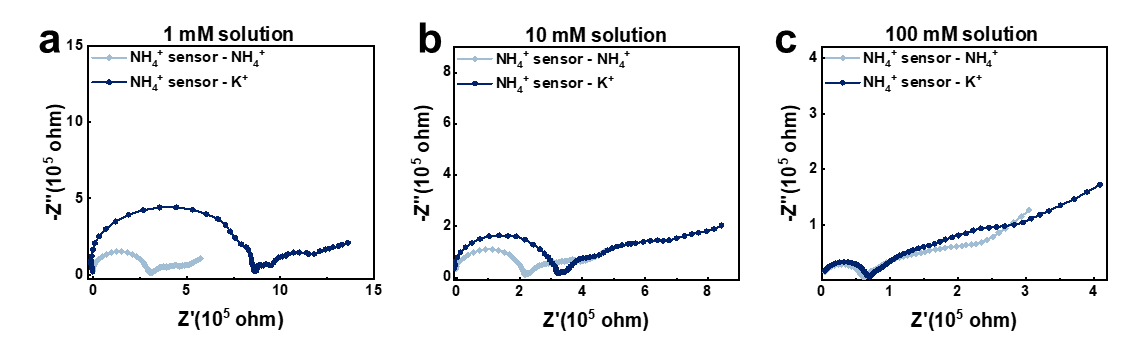


**Supplementary Fig. 7. EIS characterization of NH_4_^+^ sensor and K^+^ sensor for different concentrations. a** 1 mM NH_4_Cl. **b** 10 mM NH_4_Cl. **c** 100 mM NH_4_Cl.


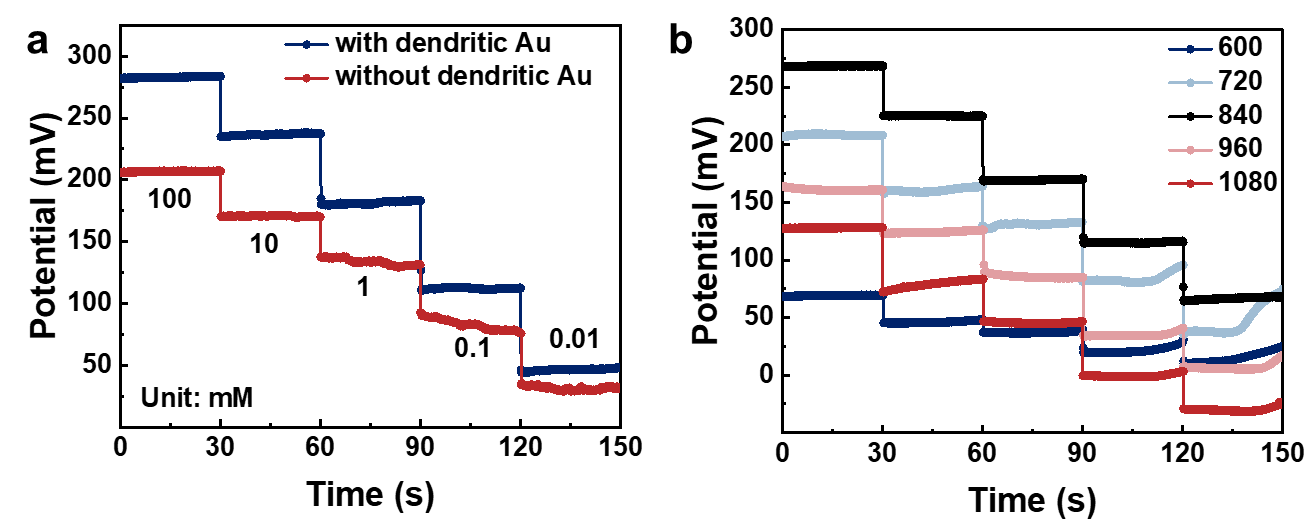


**Supplementary Fig. 8. Optimization of NH_4_^+^ sensors. a** Performance comparison of the sensors with and without dendritic Au. **b** PEDOT: PSS mass loading optimization with different deposition cycle numbers.


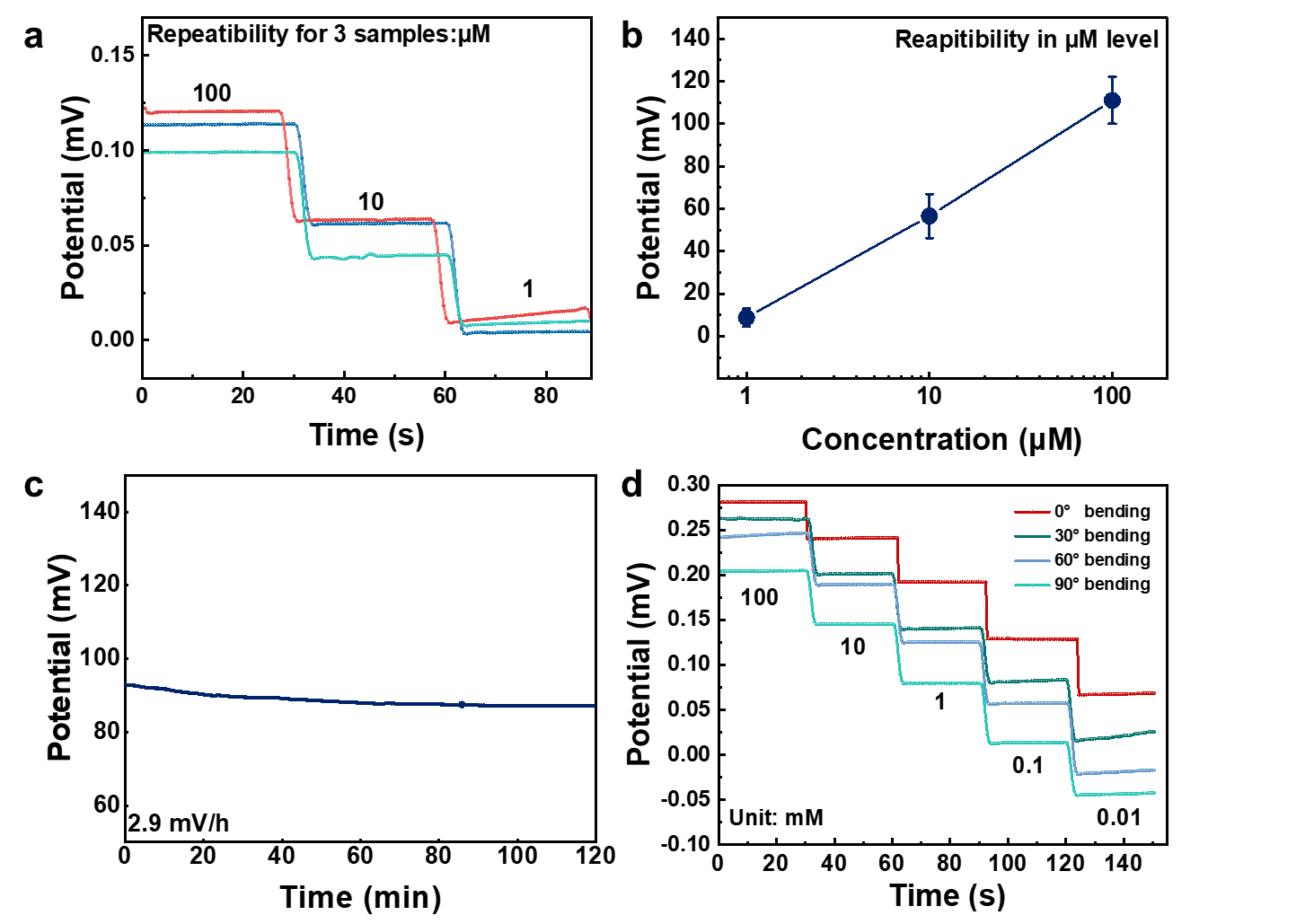


**Supplementary Fig. 9. Characterization of the NH_4_^+^ ion-selective sensors. a&b** The repeatability in the range of 1 μM - 100 μM with a sensitivity of 51 mV/decade. **c** Potential drift of NH_4_^+^ sensor in 0.05 mM NH_4_Cl solution. **d** The sensor performance in different bending angle: 0°, 30°, 60° and 90°.

**
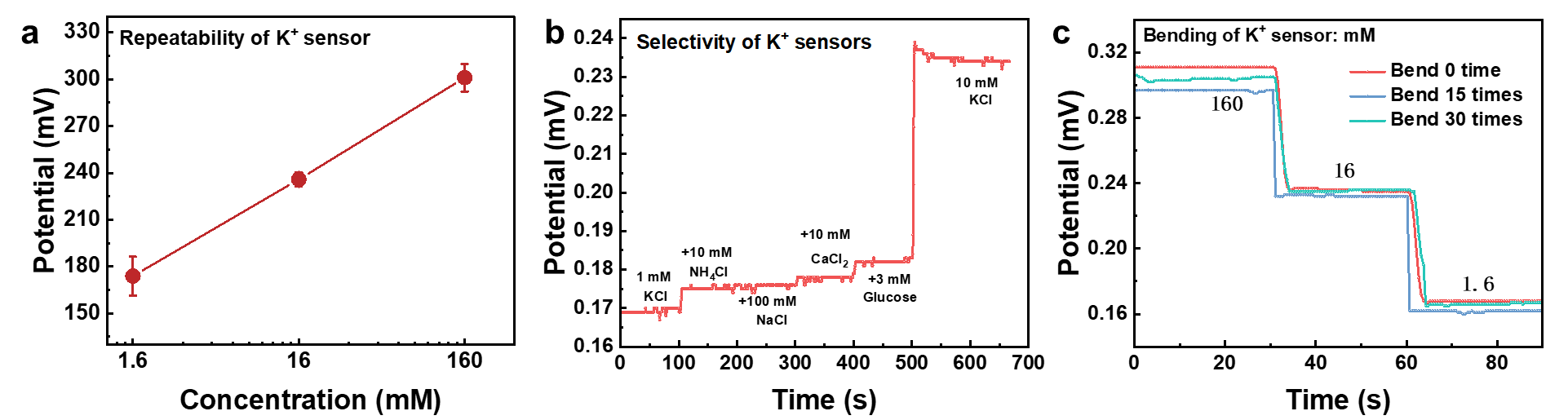
**

**Supplementary Fig. 10. Optimization of K^+^ sensors. a** Sensing performance in the range of 1.6 mM - 160 mM with sensitivity of 64 mV/decade. **b** Selectivity including interference biomarkers NH_4_Cl, NaCl, CaCl_2_ and glucose. **c** Bending stability after different bending times.

**
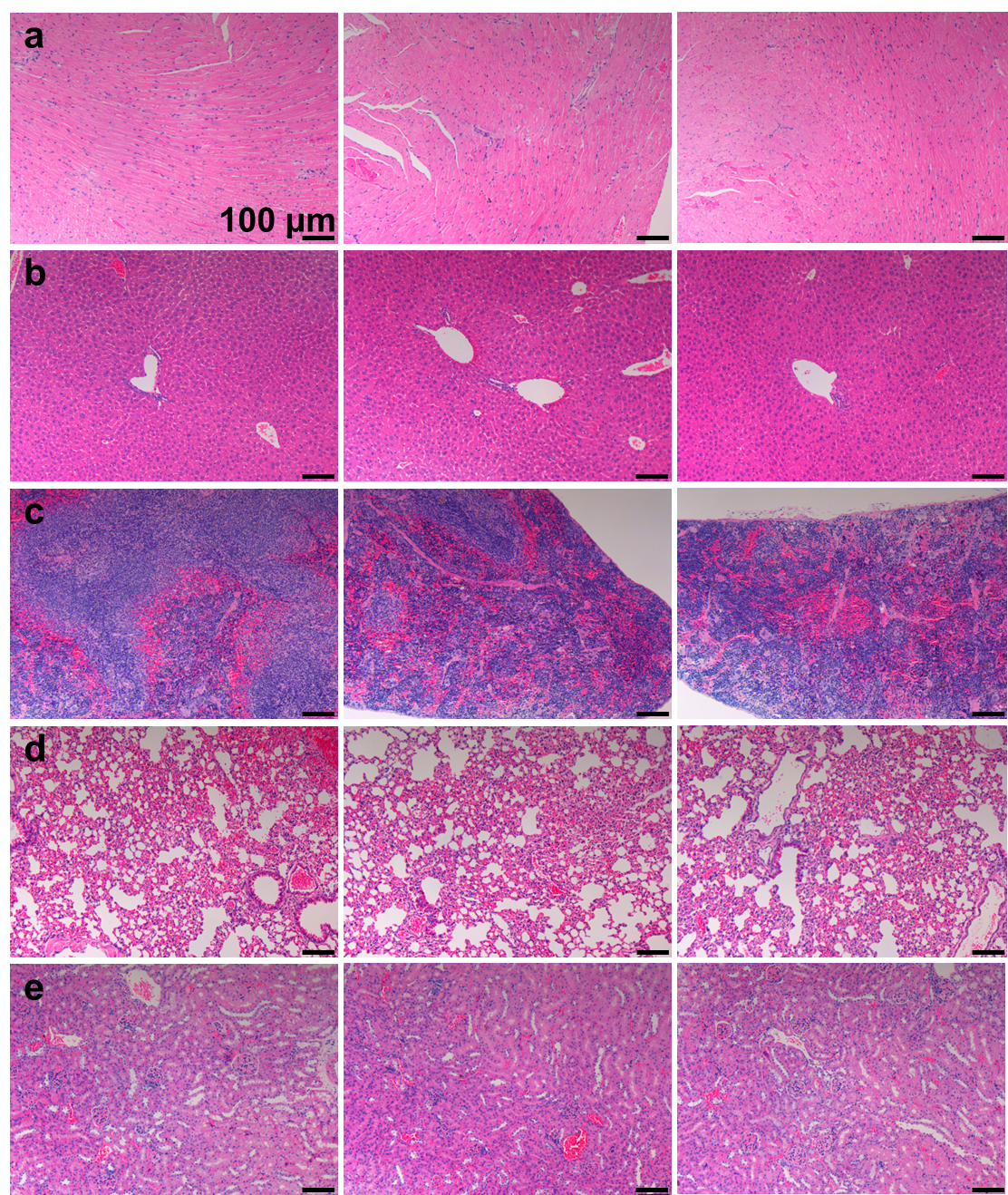
**

**Supplementary Fig. 11. *In vivo* cytotoxicity: HE staining of other muscle tissues. a** Heart. **b** Liver. **c** Spleen. **d** Lung. **e** Kidney.


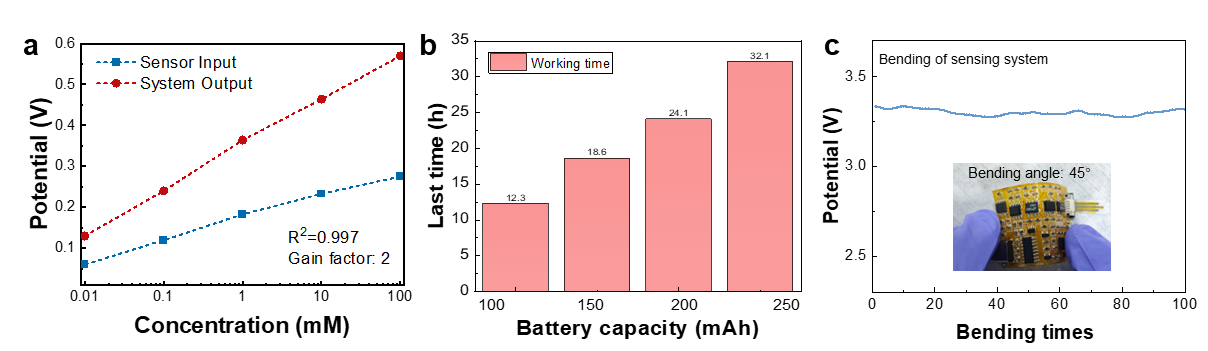


**Supplementary Fig. 12. Characterization of the sensing system. a** The OCP of the NH_4_^+^ sensor as input for the signal processing module and processed output verified with artificial sweat. **b** The long-term stability of the sensing system when using batteries with different capacities. **c** The flexibility of the sensing system.


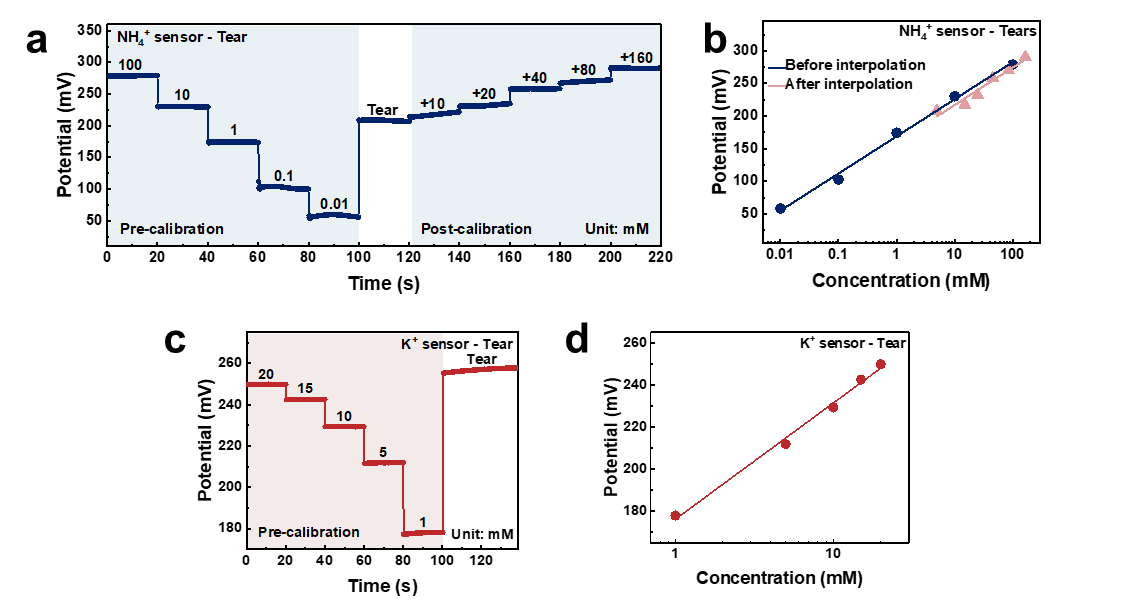


**Supplementary Fig. 13. The ammonium concentration in tears. a** The OCP response of tears with NH_4_^+^ sensor. **b** Sensitivity before and after tears interpolation. The concentration of NH_4_^+^ in tears was initially measured to be 4.93 mM and it was determined to be 1.74 mM after cross-calibration. **c** The OCP response of tears with K^+^ sensor. **d** Sensitivity of K^+^ sensor. The concentration of K^+^ in tears is 28.89 mM.


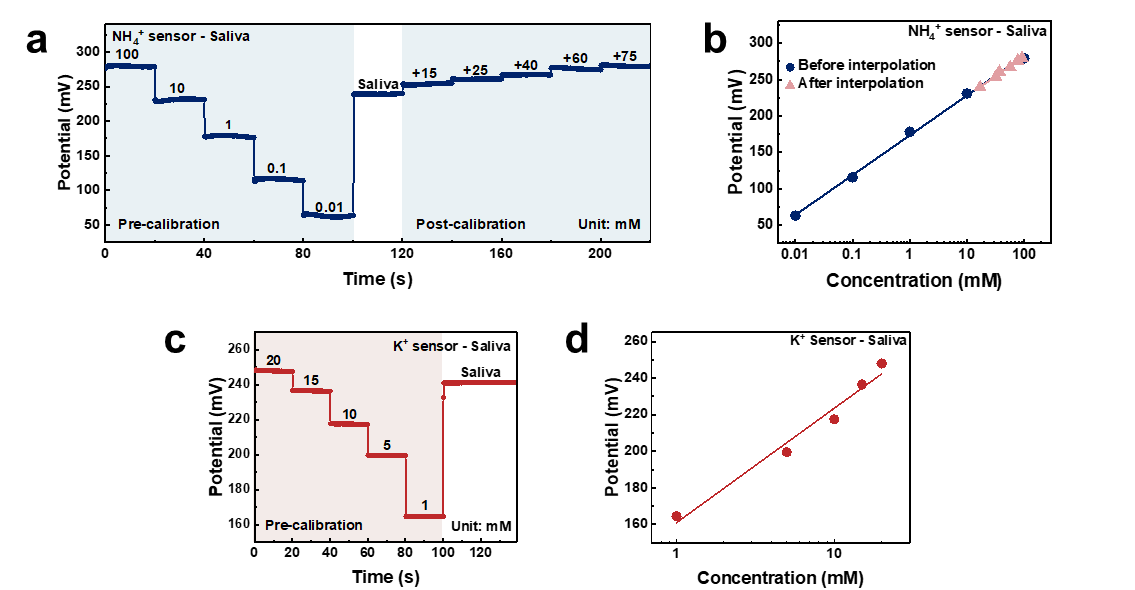


**Supplementary Fig. 14. The ammonium concentration in saliva. a** The OCP response of saliva with NH_4_^+^ sensor. **b** Sensitivity before and after saliva interpolation. The concentration of NH_4_^+^ in saliva was initially measured to be 15.89 mM and it was determined to be 13.78 mM after cross-calibration. **c** The OCP response of saliva with K^+^ sensor. **d** Sensitivity of K^+^ sensor. The concentration of K^+^ in saliva is 19.17 mM.


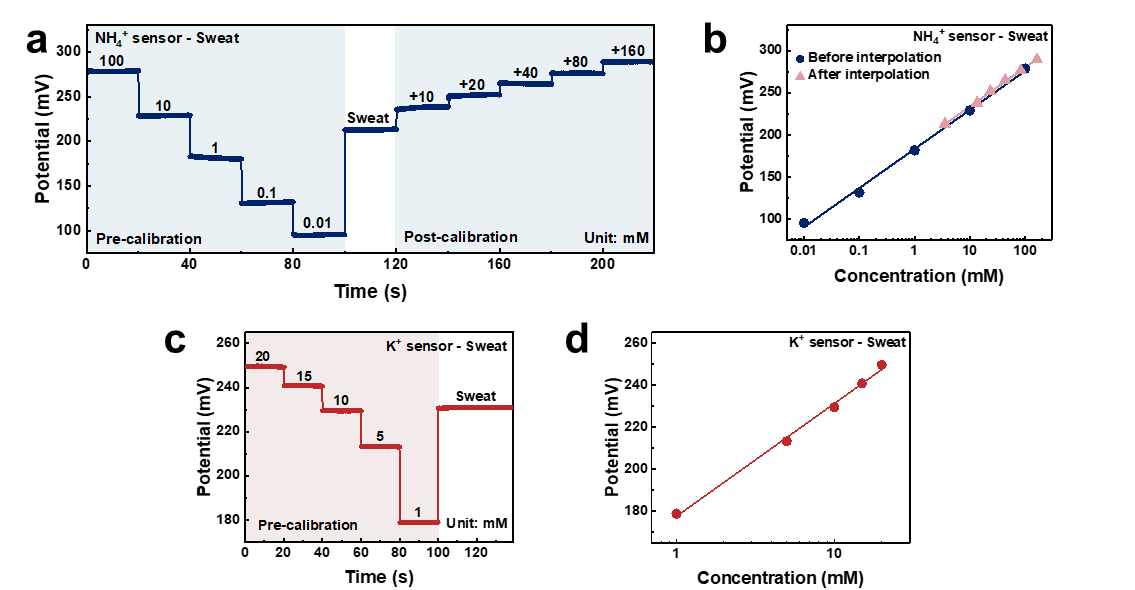


**Supplementary Fig. 15. The ammonium concentration in sweat. a** The OCP response of sweat with NH_4_^+^ sensor. **b** Sensitivity before and after sweat interpolation. The concentration of NH_4_^+^ in sweat was initially measured to be 4.39 mM and it was determined to be 3.30 mM after cross-calibration. **c** The OCP response of sweat with K^+^ sensor. **d** Sensitivity of K^+^ sensor. The concentration of K^+^ in sweat is 9.88 mM.


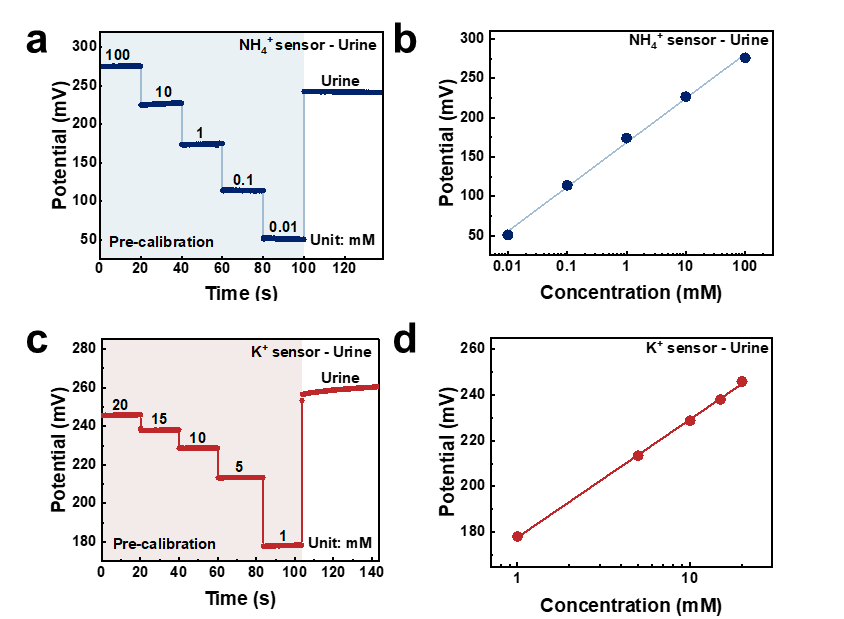


**Supplementary Fig. 16. The ammonium concentration in urine. a** The OCP response of urine with NH_4_^+^ sensor. **b** Sensitivity before sweat interpolation. The concentration of NH_4_^+^ in urine was initially measured to be 20.52 mM and it was determined to be 16.40 mM after cross-calibration. **c** The OCP response of urine with K^+^ sensor. **d** Sensitivity of K^+^ sensor. The concentration of K^+^ in urine is 37.59 mM.


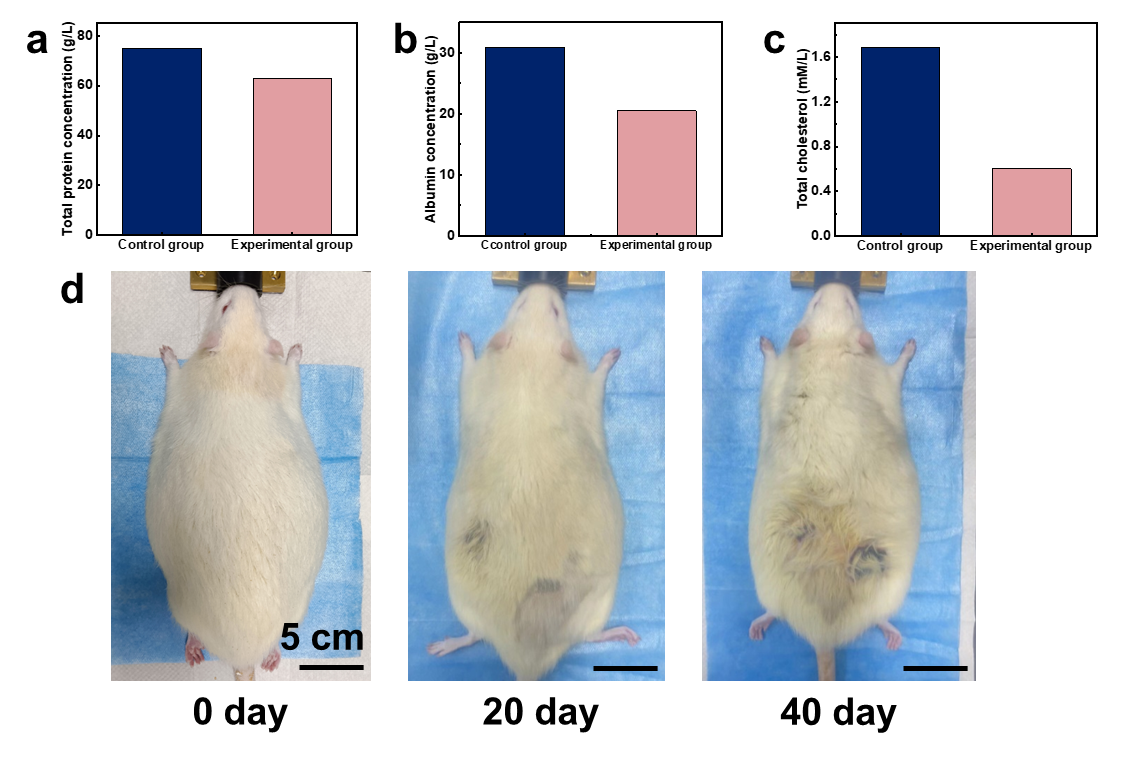


**Supplementary Fig. 17. Modeling of cirrhotic mice with morphological changes in mice**. **a** Total protein concentration. **b** Albumin concentration. **c** Total cholesterol. **d** Morphological changes in mice - day 0, day 20, day 40.


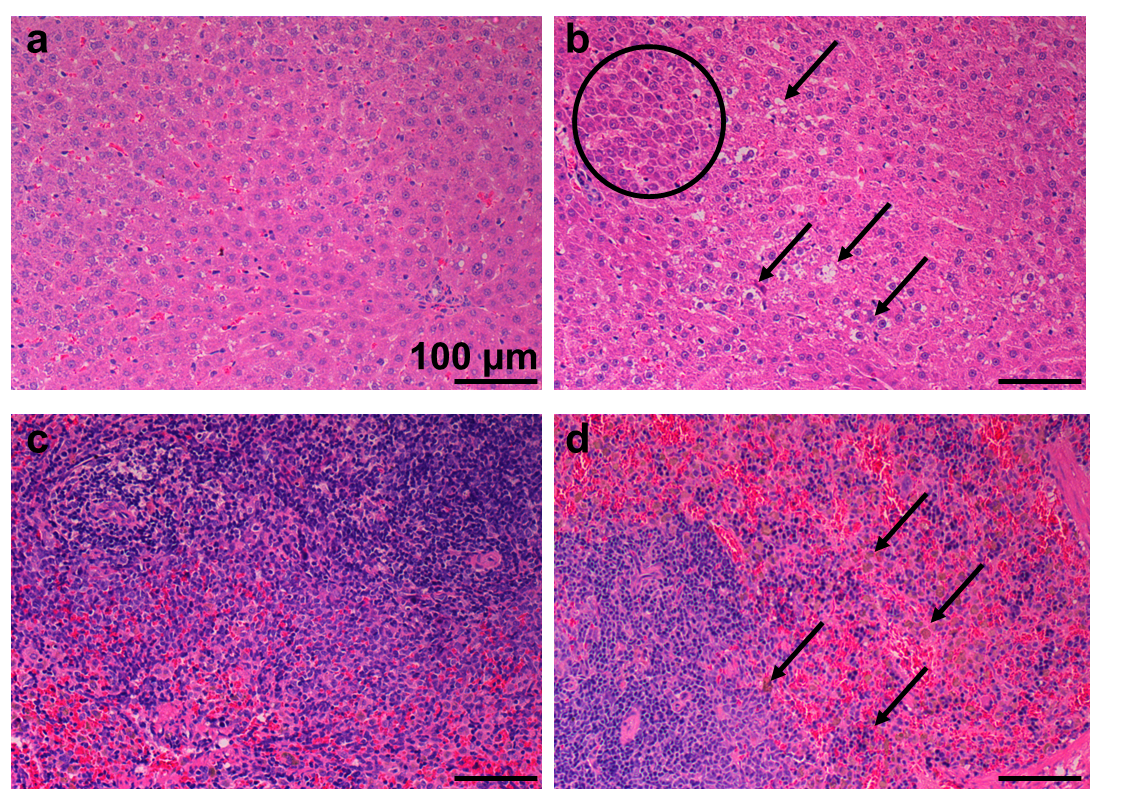


**Supplementary Fig. 18. Inflammation of liver cirrhosis modeling cells. a** Healthy mouse liver slice. **b** Liver slices from mice in the liver cirrhosis model exhibiting notable features such as collagen deposition, the formation of fibrotic tissue masses, and the aggregation of lipid droplets. **c** Healthy mouse spleen slices. **d** Spleen slices from mice in the liver cirrhosis model revealing discernible alterations in cell nucleus size, division, and staining depth.


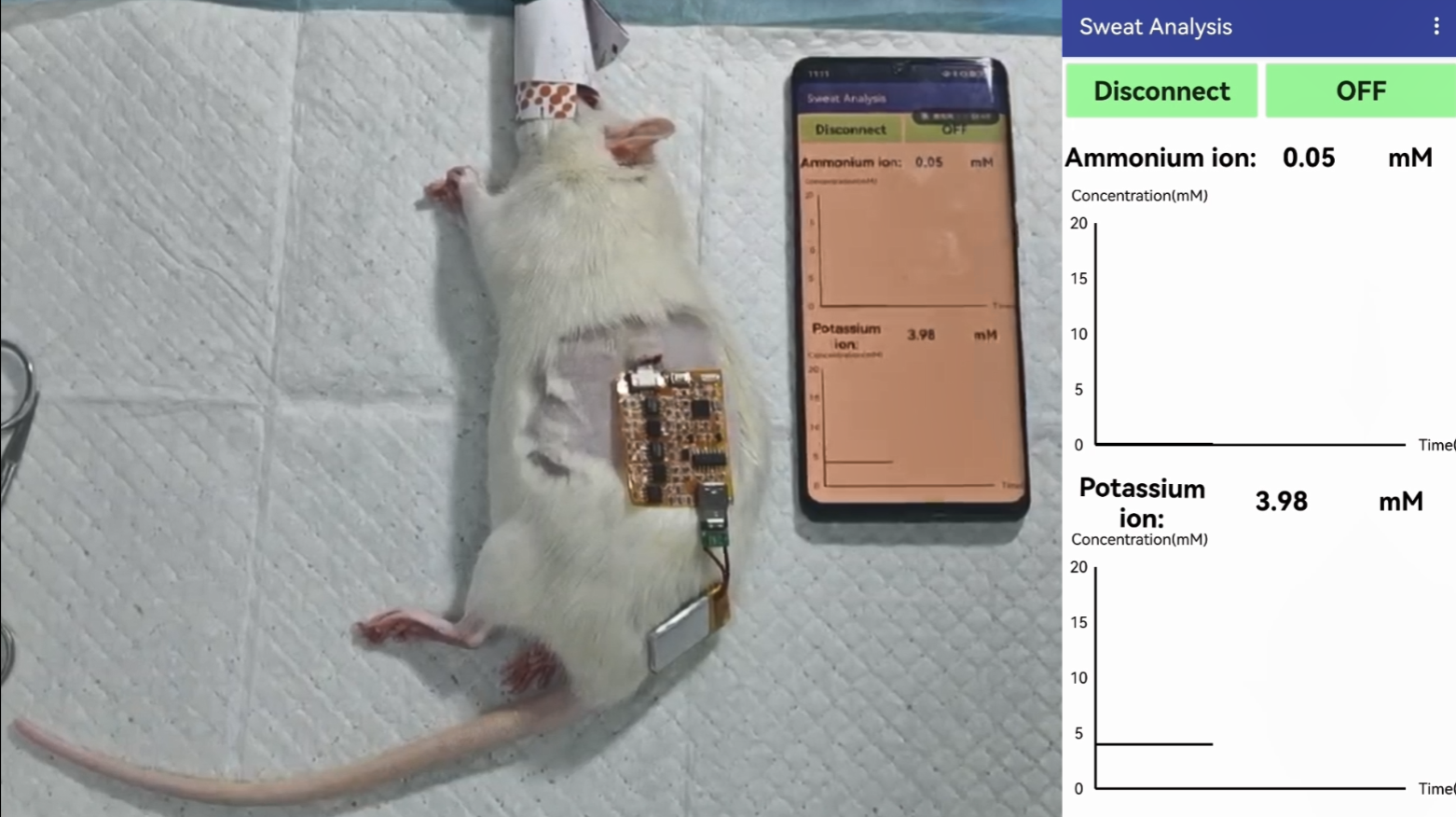


**Supplementary Fig. 19. *In vivo* demonstration of the integrated and wireless biosensing patch for continuously NH_4_^+^ concentration monitoring.**


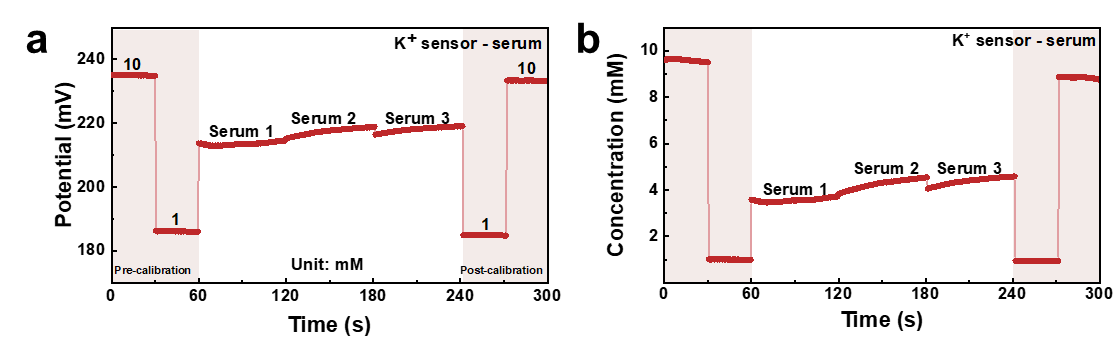


**Supplementary Fig. 20. The calculated results of blood K^+^ concentration. a** The OCP response of Blood K^+^. **b** The concentration of blood K^+^.

**
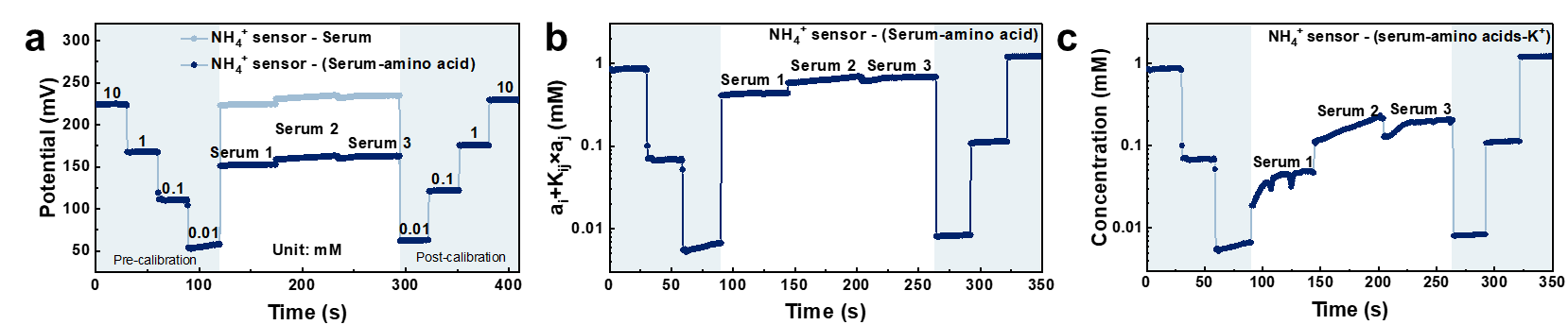
**

**Supplementary Fig. 21. The cross-calibration and calculated results of blood NH_4_^+^ concentration. a** The OCP response of blood NH_4_^+^ before and after calibrating the influence of amino acids. **b** The extracted NH_4_^+^ concentrations without cross-calibration of K^+^. **c** The concentration of Blood NH_4_^+^.

**Supplementary Table 1. Various potentiometric sensors applied for ammonium detection in the last 10 years.**

| Sensing element/structure | Sensitivity (mV) | Selectivity  (logKij)* | Detection range  (mM) | Detection limit  (μM) | Interference elimination | Application | Ref. |
| --- | --- | --- | --- | --- | --- | --- | --- |
| Nonactin | 59.5 | -0.9 | 0.01-1 | 1 | No | Sea water | ^1^ |
| Nonactin | 54.2 | -0.6 | 0.1-100 | 10 | No | Agricultural water | ^2^ |
| Nonactin | 52.9 | -0.85 | 0.1-10 | 4 | No | Tap and well water | ^3^ |
| Nonactin | 57.0 | -0.8 | 0.01-100 | 1 | No | Human urine | ^4^ |
| Nonactin | 55.3 | - | 2.5-40 | 500 | No | Sweat | ^5^ |
| Nonactin | 53.0 | -0.81 | 0.1-150 | 100 | No | Urine | ^6^ |
| Tripodal Tris(pyrazolyl) Compounds | 63.0 | -0.74 | 0.01-10 | 10 | No | Sea water | ^7^ |
| Poly (o-phenylenediamine) | 55.7 | -0.94 | 0.02-100 | 12 | No | rivers, lakes and industrial sewage | ^8^ |
| Thiazole benzo-crown ether ethylamine-thioctic acid | 53.0 | -1.1 | 0.01-100 | 10 | No | - | ^9^ |
| Prussian blue analogue of  Cu (II)-hexacyanoferrate | 56.0 | -1.1 | 0.01-100 | 42 | No | - | ^10^ |
| **Nonactin** | **58.7** | **-0.96** | **0.01-100** | **1** | **Yes** | **Universal body fluids** | **This work** |

* The smaller the selectivity coefficient, the better the selectivity

**Supplementary Table 2. Common concentrations of NH_4_^+^ and K^+^ in different body fluids**

| Body fluid | Human Blood | Mice blood | Tear | Saliva | Sweat | Urine |
| --- | --- | --- | --- | --- | --- | --- |
| NH_4_^+^ | 18-72 μM^11^ | 13-63 μM^12^ | - | 7-16 mM^13^ | 1-8 mM^14^ | 15-56 mM^15^ |
| K^+^ | 3.5-5.5 mM^11^ | 3.1-6.1 mM^16^ | 15-35 mM | 10-36 mM | 2-8 mM^14^ | 25-125 mM^15^ |

Supplementary Table 3. Human and mice plasma amino acid concentrations

| Amino acid | Human average value (μM) | Human range (μM) | Ref. | Mice average value(μM) | Ref. |
| --- | --- | --- | --- | --- | --- |
| Alanine | 333 | S.D. 74 | ^17^ | 370 | ^18^ |
| Arginine | 80 | S.D. 20 |  | 85 |  |
| Citrulline | 38 | S.D. 8 |  | 58 |  |
| Glutamic acid | 24 | S.D. 15 |  | 70 |  |
| Glutamine | 586 | S.D. 84 |  | 457 |  |
| Glycine | 230 | S.D. 52 |  | 254 |  |
| Histidine | 82 | S.D. 10 |  | 55 |  |
| Leucine | 123 | S.D. 25 |  | 78 |  |
| Lysine | 188 | S.D. 32 |  | 181 |  |
| Methionine | 25 | S.D. 4 |  | 39 |  |
| Phenylalanine | 57 | S.D. 9 |  | 45 |  |
| Threonine | 140 | S.D. 33 |  | 112 |  |
| Tyrosine | 59 | S.D. 12 |  | 47 |  |
| Valine | 233 | S.D. 43 |  | 85 |  |

**Supplementary Table 4. Comparison of different ions and molecular radii.**

| Ionic/molecular | Ammonium | Potassium | Calcium | Sodium | Glucose |
| --- | --- | --- | --- | --- | --- |
| Radius (pm) | 143^19^ | 138^20^ | 100^21^ | 102^19^ | 361^22^ |

**Supplementary Table 5. Summary of NH_4_^+^ concentration in body fluids.**

| Body fluid | Healthy mouse serum 1 | Liver cirrhosis mouse serum 2 | Liver cirrhosis mouse serum 3 | Healthy human tears | Healthy human sweat | Healthy human saliva | Healthy human urine |
| --- | --- | --- | --- | --- | --- | --- | --- |
| Normal value | 13-63  μM^19^ | Greater than 144 μM | Greater than 144 μM | - | 1-8  mM | 7-16  mM | 15-56  mM |
| Ammonium ion results after cross-calibration | 36.86  μM | 166.28  μM | 179.39  μM | 1.74  mM | 3.30  mM | 13.78  mM | 16.40  mM |
| Standard test results | 28.7  μM | 158.2  μM | 151.2  μM | 2.25  mM | 3.53  mM | 13.07  mM | 17.39  mM |
| Relative error | 28.43% | 5.11% | 18.64% | 22.57% | 6.42% | 5.44% | 5.72% |

**References:**

1. Ding L, Ding J, Ding B, Qin W. Solid-contact Potentiometric Sensor for the Determination of Total Ammonia Nitrogen in Seawater. *Int J Electrochem* **12**, 3296-3308 (2017).

2. Huang Y*, et al.* A novel all-solid-state ammonium electrode with polyaniline and copolymer of aniline/2, 5-dimethoxyaniline as transducers. *J Electroanal Chem* **741**, 87-92 (2015).

3. Schwarz J, Trommer K, Mertig M. Solid-Contact Ion-Selective Electrodes Based on Graphite Paste for Potentiometric Nitrate and Ammonium Determinations. *American Journal of Analytical Chemistry* **09**, 591-601 (2018).

4. Liu Y, Cánovas R, Crespo GA, Cuartero M. Thin-Layer Potentiometry for Creatinine Detection in Undiluted Human Urine using Ion-Exchange Membranes as Barriers for Charged Interferences. *Anal Chem*, (2020).

5. Yu Y*, et al.* Biofuel-powered soft electronic skin with multiplexed and wireless sensing for human-machine interfaces. *Sci Robot* **5**, eaaz7946 (2020).

6. Kucherenko IS*, et al.* Ion‐selective sensors based on laser‐induced graphene for evaluating human hydration levels using urine samples. *Adv Mater Technol-US* **5**, 1901037 (2020).

7. Colozza N*, et al.* Insights into tripodal tris (pyrazolyl) compounds as ionophores for potentiometric ammonium ion sensing. *ChemElectroChem* **9**, e202200716 (2022).

8. Kan Y*, et al.* An all-solid-state ammonium ion-selective electrode based on polyaniline as transducer and poly (o-phenylenediamine) as sensitive membrane. *Int J Electrochem* **11**, 9928-9940 (2016).

9. Jin S*, et al.* Voltammetric ion-channel sensing of ammonium ion using self-assembled monolayers modified with ionophoric receptors. *Sensor Actuat B-chem* **207**, 1026-1034 (2015).

10. Xu L*, et al.* Beyond nonactin: potentiometric ammonium ion sensing based on ion-selective membrane-free Prussian blue analogue transducers. *Anal Chem* **94**, 10487-10496 (2022).

11. Blood Krebs H. Chemical composition of blood plasma and serum. *Annu Rev Biochem* **19**, 409-430 (1950).

12. Koizumi T, Hayakawa J, Nikaido H. Blood ammonia concentration in mice: normal reference values and changes during growth. *Lab Anim Sci* **40**, 308-311 (1990).

13. Bhogadia M, Edgar M, Hunwin K, Page G, Grootveld M. Detection and quantification of ammonia as the ammonium cation in human saliva by 1H NMR: a promising probe for health status monitoring, with special eeference to cancer. *Metabolites* **13**, 792 (2023).

14. Baker LB. Physiology of sweat gland function: The roles of sweating and sweat composition in human health. *Temperature* **6**, 211-259 (2019).

15. Sarigul N, Korkmaz F, Kurultak İ. A new artificial urine protocol to better imitate human urine. Sci Rep 9: 20159.) (2019).

16. Boehm O*, et al.* Clinical chemistry reference database for Wistar rats and C57/BL6 mice. *Biol Chem* **388**, 547-554 (2007).

17. Cynober LA. Plasma amino acid levels with a note on membrane transport: characteristics, regulation, and metabolic significance. *Nutrition* **18**, 761-766 (2002).

18. Takach E, O'Shea T, Liu H. High-throughput quantitation of amino acids in rat and mouse biological matrices using stable isotope labeling and UPLC–MS/MS analysis. *J Chromatogr* **964**, 180-190 (2014).

19. Epsztein R, Shaulsky E, Qin M, Elimelech M. Activation behavior for ion permeation in ion-exchange membranes: Role of ion dehydration in selective transport. *J Membr Sci* **580**, 316-326 (2019).

20. Qiu H*, et al.* SnO 2 nanoparticles anchored on carbon foam as a freestanding anode for high performance potassium-ion batteries. *Energ Environ Sci* **13**, 571-578 (2020).

21. Sugiura Y, Saito Y, Endo T, Makita Y. Effect of the ionic radius of alkali metal ions on octacalcium phosphate formation via different substitution modes. *Cryst Growth Des* **19**, 4162-4171 (2019).

22. Chen K-C, Li Y-L, Wu C-W, Chiang C-C. Glucose sensor using U-shaped optical fiber probe with gold nanoparticles and glucose oxidase. *Sensors* **18**, 1217 (2018).
